# Mechanism, Efficacy, and Safety of Natural Antibiotics

**DOI:** 10.3390/antibiotics14100981

**Published:** 2025-09-29

**Authors:** Andrei Teodor Matei, Anita Ioana Visan

**Affiliations:** 1 IT Center for Science and Technology, 011702 Bucharest, Romania; matei.andrei5@gmail.com; 2 National Institute for Lasers, Plasma and Radiation Physics, 077125 Magurele, Romania

**Keywords:** antimicrobial resistance, antibiotic alternatives, drug-delivery systems, drug-resistant bacteria, antimicrobial strategies

## Abstract

The growing ineffectiveness of common antibiotics against multidrug-resistant pathogens has made antimicrobial resistance (AMR) a serious global health concern. This review emphasizes that natural antibiotics from animals, bacteria, fungi, and plants are worthy alternatives for combating this crisis. Evolutionary pressure has shaped these molecules, leading to antibiotic-resistant bacteria that can withstand single-target synthetic drugs but are vulnerable to multiple attack pathways (e.g., cell wall disruption, protein synthesis inhibition, biofilm interference) from natural compounds. Natural antibiotics are frequently incorporated into treatment strategies or drug-delivery systems for minimizing side effects, reducing doses, and improving their effectiveness. The review discusses recent progress in this field, describing the mechanisms of action of natural antibiotics, their incorporation into several drug-delivery systems, and their ‘omics’-driven discovery to improve production, while expressing the challenges that remain. Extracellular application of these compounds, however, is compromised by their low stability in the extracellular environment; furthermore, formulation advancements, such as nanoparticle encapsulation, have been shown to enhance the bioavailability and activity of these substances. Combining indigenous knowledge and modern scientific advances, natural antibiotics may be developed to fight AMR both as monotherapy and adjuvants in a sustainable way. Leveraging these synergies, alongside the latest advances in research, is key to bridging the antibiotic discovery–resistance gap and may provide a route to clinical translation and global AMR control. The promise of natural antibiotics is clear, but their path to mainstream medicine is fraught with obstacles like reproducibility, standardization, and scalability. It is more realistic to see these substances as powerful complements to existing therapies, not outright replacements. Their true strength is in their ability to interfere with resistance mechanisms and create new possibilities for drug development, positioning them as a vital, though complicated, part of the global effort to combat AMR.

## 1. Introduction

Antimicrobial resistance (AMR) has become a significant global health challenge of the 21st century, posing a risk of undoing years of advancements in medicine [1]. O’Neill’s report (2016) estimated that, if current trends persist, antimicrobial resistance could be responsible for up to 10 million deaths annually by 2050, thus surpassing cancer as the leading global cause of mortality [2,3]. This concerning forecast highlights the critical necessity for creative strategies to address multidrug-resistant (MDR) bacteria, especially the ESKAPE pathogens (*Enterococcus faecium*, *Staphylococcus aureus*, *Klebsiella pneumoniae*, *Acinetobacter baumannii*, *Pseudomonas aeruginosa*, *and Enterobacter* spp.) [4]. Bacteria employ multiple strategies to evade the effects of antibiotics. Among the most common are the production of enzymes such as β-lactamases, the activation of efflux pumps that expel drugs from the cell, alterations in target sites like ribosomal subunits, and the formation of biofilms that provide collective protection. Each of these mechanisms reduces antibiotic efficacy and contributes to the persistence of resistant strains [5,6].

**Figure 1:**
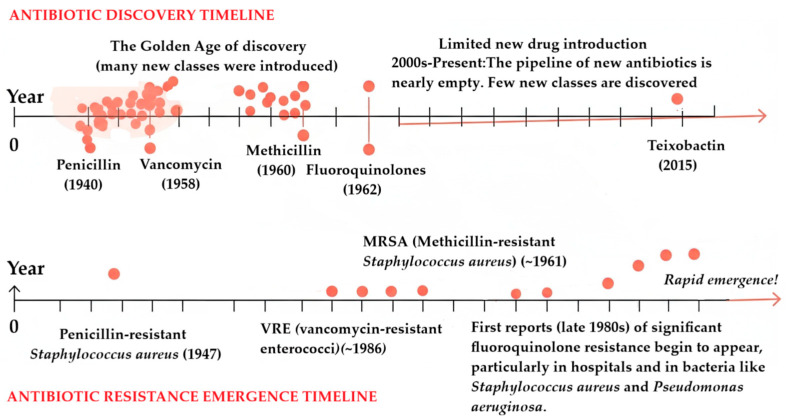
Justification of the necessity of this review: the alarming disparity between the pace of antibiotic discovery and the rapid emergence of antibiotic resistance. The timeline for new drug development is clearly insufficient to counter the escalating threat, highlighting a critical gap in addressing the global challenge of antimicrobial resistance.

The rise of AMR is a complex issue fueled by both bacterial genetic adaptability and human actions. Bacterial resistance is driven by mutations, horizontal gene transfer, and environmental reservoirs of resistance genes [7,8]. These natural processes are exacerbated by the overuse of antibiotics in both agriculture [9] and healthcare [10], inadequate infection control practices, and a stalled pipeline for new drug discovery [11]. Pharmaceutical investment in antibiotic development has declined due to economic disincentives, creating a critical gap between the rapid emergence of resistance and the slow pace of new drug discovery (Figure 1) [12]. This disparity highlights the pressing need for novel therapeutic approaches [13].

In light of these challenges, natural antibiotics—derived from plants, fungi, and bacteria—have regained prominence as promising alternatives or adjuncts to conventional therapies [14]. These compounds, shaped by millennia of evolutionary pressure, often target multiple bacterial pathways simultaneously (e.g., cell wall disruption, protein synthesis inhibition, and biofilm interference), reducing the likelihood of resistance [15,16]. Historical successes like penicillin and streptomycin highlight their potential [17], yet challenges remain. Many natural antimicrobial compounds, such as allicin from garlic [18] and berberine from barberry plants [19], can effectively inhibit a wide range of bacteria in lab settings [20]. However, it is often challenging to use them in medicine because issues arise in how the body absorbs them, they are not stable, and they can be toxic [14]. Sustainability concerns also arise from the large-scale harvesting of medicinal species [15].

This review comprehensively evaluates the mechanisms, efficacy, and safety of natural antibiotics, emphasizing their role in addressing AMR. We analyze advances in extraction, formulation (e.g., nanoparticle encapsulation) [21], and synergistic combinations to enhance therapeutic potential [22]. Furthermore, we explore how cutting-edge technologies—such as Clustered Regularly Interspaced Short Palindromic Repeats (CRISPR)-based strain engineering [23] and omics-driven discovery [24]—can optimize production and overcome limitations. By synthesizing the current evidence, this work aims to bridge the gap between traditional knowledge and modern science, offering actionable insights for developing sustainable, resistance-proof antimicrobial strategies.

**Figure 2:**
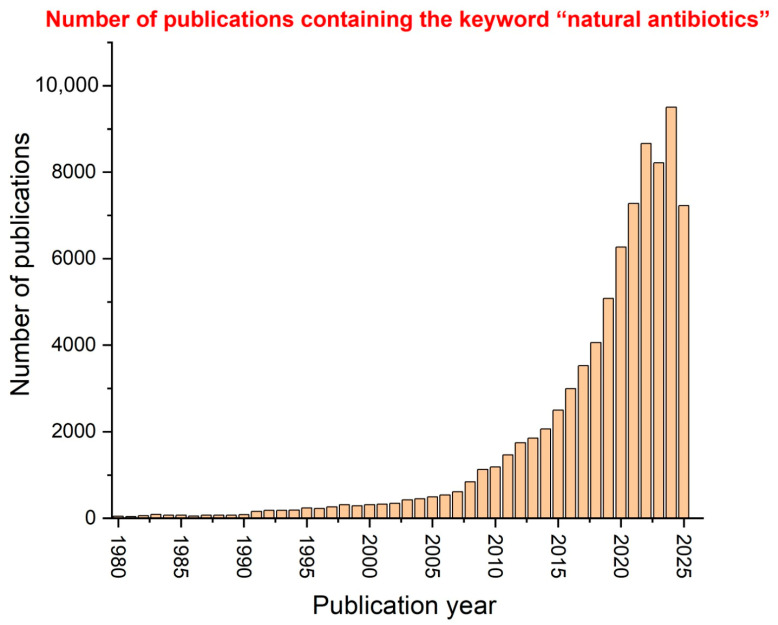
Numbers of publications on natural antibiotics per year. This analysis is based on keyword counts from the Web of Science Core Collection. The figure reflects data retrieved on 19 September 2025.

Several studies have focused on natural antibiotics in the past 45 years, and the numbers of publications containing the keyword “natural antibiotics”, published each year from 1980 to 2025, are presented in Figure 2. The data source for this graph is Web of Science, one of the most reputable and accurate electronic databases. It is observed that the peak of interest in natural antibiotics among researchers was reached between 2022 and 2024, suggesting that this subject is currently a focus area for many researchers and that the future holds great promise for further studies and innovations.

In conclusion, this review underscores the potential of natural antibiotics, but a closer look shows that their integration into mainstream medicine is not a simple process. Significant hurdles, including ensuring reproducibility, standardization, and scalability, need to be overcome. A more balanced perspective suggests that these compounds will not necessarily replace current drugs, but could instead serve as powerful complements to them. Their primary benefit is their capacity to counteract AMR mechanisms and pave the way for new drug discoveries. Therefore, they represent an important, albeit complex, part of the global effort to combat AMR.

## 2. Natural Antibiotics Classification

Natural products, especially the secondary metabolites they produce, are an incredibly diverse group of compounds with a wide range of biological functions [25]. Although these molecules are not strictly necessary for an organism’s survival in a lab, they give it a major competitive edge in the wild. Since the discovery of penicillin, over 23,000 new natural compounds have been identified, proving to be a valuable resource for medicine, agriculture, and industry [15].

**Figure 3:**
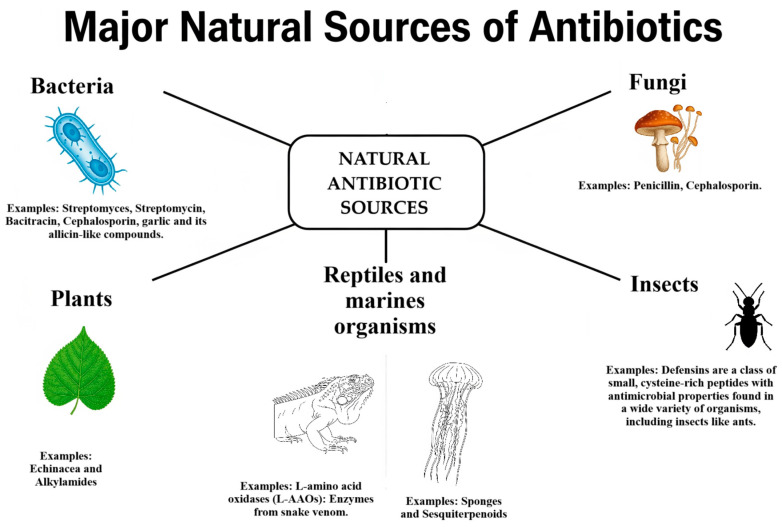
Natural antibiotics classification according to their biological origins, with five main sources: bacteria, fungi, plants, insects, reptiles, and marine organisms. For instance, fungi produce compounds such as penicillin and cephalosporins, while insects synthesize peptides like defensins, and plants generate various antimicrobial metabolites.

To support a better understanding of the diverse origins of natural antibiotics, Figure 3 presents a clear classification based on their source organisms.

### 2.1. Animal-Derived Antimicrobials

Animals have lived alongside countless microorganisms for millions of years, leading them to develop sophisticated defense mechanisms and become a rich source of various antimicrobial compounds [26]. Recent studies have focused on finding the molecules that allow some animals, like cockroaches, to survive in highly polluted, pathogen-rich environments [27].

Since their discovery in 1974, more than 150 antimicrobial peptides (AMPs) have been recognized [28]. These peptides are mainly cationic and consist of 20 to 50 amino acids, primarily functioning by disrupting bacterial plasma membranes via the formation of pores or ion [29,30]. Beyond their antibacterial properties, some AMPs also demonstrate antifungal [31], antiparasitic [32], or antiviral activities [33]. AMPs fall into four main structural groups, each with a different way of working [34]. Alpha-helical peptides, like cecropin, are effective against both Gram-positive and Gram-negative bacteria [35]. Cysteine-rich peptides, such as insect defensins, mainly target Gram-positive bacteria [36]. Proline-rich peptides, including lebocins, are active against both types of bacteria and some fungi [37]. Lastly, glycine-rich peptides, like attacin, are specifically effective against Gram-negative bacteria such as Escherichia coli [38,39]. While AMPs hold significant promise for medical therapies, further research is needed to enhance their potency and stability [34]. Their intrinsic antimicrobial capacity can be augmented through peptide fusion, as demonstrated by a hybrid peptide combining attacin from *Spodoptera exigua* [40] and a coleoptericin-like protein from *Protaetia brevitarsis seulensis* [41], which exhibited enhanced antimicrobial activity against *E. coli* [38].

#### 2.1.1. Insect-Derived Compounds

Extracts from the brain of cockroaches (*Periplaneta americana*) [42] have shown significant antimicrobial activity against methicillin-resistant *Staphylococcus aureus* (MRSA) and neuropathogenic *E. coli* K1 [43]. Although not all components were precisely identified, the extracts contained known bioactive molecules, including isoquinolines [44], flavanones [45], sulfonamides [46], and imidazones [47]. It is hypothesized that the constitutive expression of this “antimicrobial cocktail” in the cockroach brain protects its central nervous system in pathogen-rich environments [29]. Another compelling example comes from the secretions of *Lucilia cuprina* blowfly maggots [48]. Even with mild bacterial growth-inhibiting properties on their own, the secretions from these blowfly maggots—which contain defensins and phenylacetaldehyde—significantly boosted the effectiveness of the antibiotic ciprofloxacin against MRSA and slowed the development of resistance [49]. This shows the potential of using maggot debridement therapy to treat wounds infected with hospital-acquired MRSA [50].

#### 2.1.2. Bee Products

Honey exhibits antimicrobial activity against both Gram-negative bacteria (e.g., *E. coli*, *Pseudomonas aeruginosa*) and Gram-positive bacteria (e.g., *Bacillus subtilis*, *S. aureus*, including MRSA) [51]. The effectiveness of honey as an antimicrobial agent is due to a combination of factors, including hydrogen peroxide [52], bee defensin-1 [53], and methylglyoxal [54]. The specific amount and type of these molecules can differ between honey varieties, which in turn influences their unique antibacterial properties and mechanisms of action [55]. Propolis, a resinous substance collected by honeybees, has been used since ancient times for its biological properties. Its antimicrobial activity against *S. aureus* and *E. coli* is primarily due to flavonoids, terpene derivatives, and phenolic acids, though its composition varies geographically [56]. Royal jelly, a substance made by worker bees, contains several antimicrobial compounds like royalisin [54] and jelleines [57], as well as 10-hydroxy-2-decenoic acid (10-HDA) [58]. These substances are effective against both Gram-positive and Gram-negative bacteria, including drug-resistant strains such as MRSA [55]. Melittin, a major component of Apis mellifera bee venom, has also demonstrated promising antimicrobial activity, including in vivo efficacy against MRSA in mouse models [59].

#### 2.1.3. Reptile and Marine Animal Compounds

Reptiles like snakes, which can eat germ-infected rodents without becoming sick, are a great source of new antimicrobial molecules. For example, studies on the plasma of the Black cobra (Naja naja karachiensis) found that it had a powerful effect against *E. coli* K1 and MRSA, with an efficacy comparable to the antibiotic gentamicin [60]. Lung and gallbladder lysates from the cobra also exhibited high antimicrobial capacity against MRSA [61]. Cathelicidin-BF, an antimicrobial peptide isolated from the venom of Bungarus fasciatus, has shown high activity against drug-resistant Gram-negative bacteria [62]. Crotalus adamanteus toxin-II (CaTx-II) demonstrated strong antimicrobial effects against *S. aureus*, *Burkholderia pseudomallei*, and *Enterobacter aerogenes* by forming membrane pores and damaging cell membranes, notably without cytotoxicity to mammalian cells in vitro or in treated mice [63]. The anti-lipopolysaccharide factor from red claw crayfish, a type of crustacean, demonstrated potent antimicrobial properties [64]. It showed a high capacity to kill bacteria, with very low minimum bactericidal concentrations against both the Gram-negative bacterium *Shigella flexneri* [65] and the Gram-positive bacterium *S. aureus* [66]. The mechanism of action for this compound appears to be independent of bacterial plasma membrane alteration, warranting further investigation. The venom of the Mexican scorpion Vaejovis mexicanus contains vejovine [67], an AMP active against MDR Gram-negative bacteria with MIC values ranging from 4.4 μM to 50 μM [68].

Research into antimicrobial peptides from animal sources reveals an intriguing aspect of evolutionary defense mechanisms [69]. Despite this potential, their transition to clinical use is hindered by several formidable obstacles, including potential immunogenicity and costly production methods [38]. The main engineering challenge is to modify these peptides to be sufficiently stable within the human body while maintaining their effectiveness at nontoxic concentrations, a significant hurdle that has yet to be fully overcome [70].

### 2.2. Bacterial Antimicrobials

Bacteria, particularly those belonging to the actinomycetes class, remain the most prolific source of natural products with antimicrobial activity discovered to date [71]. Because of their wide variety, competitive nature, and ability to colonize, bacteria have evolved secondary metabolites that give them significant advantages over other bacterial species [72]. The identification and isolation of these bacterial antimicrobial NPds were pivotal in propelling medical science forward during the mid-20th century, often referred to as the “Golden Age” of antibiotic discovery [71].

Vancomycin, a significant antibiotic, is a naturally occurring compound that was successfully extracted from the actinomycete bacterium, *Streptomyces orientalis* [73]. This tricyclic glycopeptide has proven to be highly effective against Gram-positive bacteria [74], including challenging strains like MRSA and penicillin-resistant pneumococci [75]. Its mechanism involves forming hydrogen bonds with the terminal dipeptide of the nascent peptidoglycan chain, thereby disrupting bacterial cell wall synthesis and ultimately leading to cell rupture and death [74]. Baulamycin, isolated from the marine bacterium *Streptomyces tempisquensis* [76], inhibits the biosynthesis of iron-chelating siderophores in *S. aureus* (targeting staphylopherrin B) and *Bacillus anthracis* (targeting petrobactin), offering potential for treating MRSA and anthrax infections [77]. Fasamycin A, a polyketide from the bacterium *Streptomyces albus*, specifically targets and inhibits Gram-positive bacteria like vancomycin-resistant Enterococci (VRE) and MRSA [78], with MIC values of 0.8 and 3.1 μg/mL, respectively. Separately, it also showed an ability to stop the growth of Gram-negative bacteria such as *S. flexneri* and *E. coli*, suggesting it has a broad-spectrum potential [79]. This molecule targets FabF in the initial condensation step of bacterial lipid biosynthesis. Orthoformimycin, produced by *S. griseus*, inhibits bacterial translation in *E. coli* by over 80%, possibly by decoupling mRNA and aminoacyl-tRNA in the ribosome [80]. Kibdelomycin, isolated from *Kibdelosporangium* sp., MA7385, is a potent inhibitor of DNA synthesis. Its unique structure and function as a bacterial type II topoisomerase inhibitor make it the first such natural product discovered in over 60 years [81]. It shows broad-spectrum activity against aerobic bacteria, including MRSA (MIC 0.25 μg/mL), and remarkably low rates of resistance development, comparable to successful antibiotics like ciprofloxacin [81]. Pyridomycin, from *Dactylosporangium fulvum*, demonstrates strong antimycobacterial activity, including against isoniazid-resistant *Mycobacterium tuberculosis* strains [81]. It targets the cell wall by inhibiting mycolic acid production via NADH-dependent enoyl-(Acyl-Carrier-Protein) reductase InhA, with MBC values between 0.62 and 1.25 μg/mL against *M. tuberculosis* [82].

From a bacterial source, myxovirecin is a macrocyclic compound from myxobacteria that has broad-spectrum antibacterial properties. It works by preventing the processing of a key lipoprotein, Lpp, which is essential for the bacteria to function [83]. It showed potent activity against *E. coli* DW37 with a MIC of 0.063 μg/mL. Spirohexenolide A, a natural spirotetronate from *Spirulina platensis* (cyanophyceae), disrupts the cytoplasmic membrane of methicillin-resistant *S. aureus*, collapsing its proton motive force [16]. Teixobactin, produced by *Eleftheria terrae* (betaproteobacteria), shows antibacterial capacity against antibiotic-resistant pathogens in animal infection models [84]. Its mechanism involves binding to bacterial wall teichoic acid precursors, leading to cell wall digestion by autolysins [84]. Notably, no resistance has been detected against teixobactin in its producer strain or in laboratory mutants [85]. Lipoglycopeptides, such as actinocarbasin (from *Actinoplanes ferrugineus* strain MA7383), arylomycin, and krisynomycin (from *Streptomyces fradiae* strain MA7310), inhibit signal peptidase type IB (SpsB), a membrane-localized serine protease crucial for processing secreted proteins [85]. Actinocarbasin, for instance, enhances the activity of β-lactam antibiotics against MRSA. Beyond direct antimicrobial action, some bacterial molecules target virulence factors. Skyllamycins B and C, cyclic depsipeptides from marine bacteria, inhibit and disperse *P. aeruginosa* biofilms [86]. Biofilms are a major cause of drug resistance in nosocomial infections; these molecules, while not bactericidal on their own, restore the activity of antibiotics like azithromycin in the presence of biofilms [87].

Clatworthy and colleagues (2007) emphasized that, although bacteria represent a valuable source of antimicrobial molecules, they often already possess resistance mechanisms against their own products. A more innovative research direction is the development of therapies that inhibit bacterial resistance mechanisms or virulence factors, thereby offering therapeutic benefits distinct from those of conventional antibiotics [88,89]. This approach could offer a truly novel therapeutic benefit.

### 2.3. Fungal Antimicrobials

The fungal kingdom is a vast and largely untapped resource for discovering new antibiotics, with an estimated 2.2 to 3.8 million species yet to be explored. This immense biodiversity offers significant potential for finding novel antimicrobial natural products [90].

King and co-workers (2014) reported that aspergillomarasmine A, a polyamino acid derived from Aspergillus versicolor, is capable of inhibiting metallo-β-lactamase enzymes, which are responsible for antibiotic resistance in Gram-negative bacteria, including *Enterobacteriaceae*, *Acinetobacter* spp., *Pseudomonas* spp., and *Klebsiella pneumoniae*. This compound demonstrated the potential to restore the efficacy of certain carbapenems against these pathogens [91,92]. This compound has successfully reversed meropenem resistance in mice infected with NDM-I-producing *K. pneumoniae*, restoring antibiotic sensitivity and resolving the infection [93]. Mirandamycin, a quinol of fungal origin, inhibits the growth of both Gram-negative and Gram-positive bacteria, with greater efficacy against the latter, including antibiotic-resistant strains like MRSA and carbapenemase-producing *K. pneumoniae*. Its mechanism involves disrupting bacterial sugar metabolism, interfering with fermentation and transport [94]. Various fungal extracts, including those from *Ganoderma lucidum*, *Ganoderma applanatum*, *Meripilus giganteus*, *Laetiporus sulphureus*, *Flammulina velutipes*, *Coriolus versicolor*, *Pleurotus ostreatus*, and *Panus tigrinus*, have demonstrated antimicrobial activity against Gram-positive bacteria like *S. aureus* and B. luteus in Kirby-Bauer assays [95]. These extracts are particularly noteworthy as they represent a rich source of bioactive polysaccharides and triterpenoids with broad-spectrum efficacy; however, challenges in standardizing these complex extracts for consistent potency remain a significant limitation for clinical translation. Marine fungi, particularly those cohabiting with sponges or corals, have yielded compounds active against antibiotic-resistant bacteria. Examples include lindgomycin [96] and ascosetin, isolated from *Lindgomycetae* spp. from Baltic and Antarctic Sea sponges [97], with MIC values of 5.1 μM and 3.2 μM against MRSA, respectively. Additionally, (±)-Pestalachloride D, a chlorinated benzophenone derivative from the coral-derived fungus *Pestalotiopsis* sp. [98], exhibits antibacterial activity against *E. coli*, Vibrio anguillarum, and Vibrio parahaemolyticus (MICs of 5, 10, and 20 μM, respectively) [99]. Trichodermins, aminolipopeptides from a sponge-derived *Trichoderma* sp., possess potent antimycobacterial activity *against Mycobacterium smegmatis*, *Mycobacterium bovis* BCG, and *M. tuberculosis* H37Rv, with MIC values between 0.02 and 2.0 μg/mL under various conditions [100].

A key natural antibiotic derived from a fungal source is fusidic acid, a tetracyclic triterpenoid. Originally isolated from the fungus *Fusidium coccineum*, it has been used clinically for decades, particularly against Gram-positive bacteria like methicillin-resistant *Staphylococcus aureus.* Its primary mechanism of action involves inhibiting bacterial protein synthesis by binding to and blocking elongation factor G, an essential protein in the translation process. This unique mode of action makes fusidic acid a valuable option in combating antibiotic resistance, as it has no cross-resistance with other major antibiotic classes [101].

A significant hurdle in using fungi for antibiotic production is that it is challenging to cultivate many species, and their production of metabolites, like antibiotics, is often inconsistent [102]. Rather than simply looking for new fungal compounds, the most effective path forward is to use modern genomics and synthetic biology to improve and standardize the production process. This would lead to a reliable and scalable supply of these crucial pharmaceuticals [103].

### 2.4. Plant-Derived Antimicrobials

Plants possess an immense chemical diversity, with roughly 170,000 unique secondary metabolites, making them an excellent source for new medicines. These compounds, which include alkaloids, terpenoids, and polyphenols, are naturally produced by plants to protect against various threats, including infections. This vast reservoir of biomolecules offers a promising avenue for developing new antibiotics with broad efficacy and favorable safety profile [104].

Since ancient times, humans have used plants for medicine, as seen in historical records like the Ebers Papyrus and Ayurvedic texts that mention the use of herbs like garlic, turmeric, and aloe vera. Today, modern technology allows scientists to re-examine this traditional knowledge to understand the molecular and functional properties of these plants, paving the way for new therapeutic applications [105].

Alkaloids compounds have demonstrated antimicrobial capacity against various bacterial species. While studies on pure alkaloids are limited, plant extracts rich in alkaloids have shown activity [106]. For instance, extracts from *Papaver rhoeas*, with roemerine as a main active component, exhibited activity against *S. aureus*, *Staphylococcus epidermidis*, and *K. pneumoniae* [107]. Raw alkaloid-rich extracts from Annona squamosa seeds and Annona muricata root also showed moderate antimicrobial activity against *E. coli* and *S. aureus* [108].

Terpenoids are often found as components of plant essential oils, terpenoids contribute to antimicrobial activity. Although many terpenoids may not possess significant antimicrobial activity per se, their hydrophobic nature and low molecular weight enable them to disrupt bacterial cell walls and facilitate the penetration of other active components within essential oils, thereby enhancing overall antimicrobial efficacy [109,110].

Plant molecules known as polyphenols—characterized by one or more phenolic groups—act as a defense against stress. A significant amount of research confirms that these compounds, and plant extracts rich in them, have a powerful ability to kill or inhibit the growth of many Gram-positive and Gram-negative bacteria [111]. Their potential as antimicrobials is being widely explored for applications in agriculture, food preservation, and medicine [112]. Polyphenols are classified into subfamilies based on their chemical structures, including flavonoids (e.g., anthocyanidins, flavanones, flavones, flavonols, isoflavones), hydrolyzable tannins, lignans, phenolic acids, and stilbenes. Notable flavonoids with antimicrobial activity include quercetin, kaempferol, morin, myricetin, epigallocatechin gallate (EGCG), and galangin. Punicalagin, a tannin, demonstrates both antibacterial and antibiofilm effects against *S. aureus*. Resveratrol, a stilbene, exhibits broad-spectrum antimicrobial activity, including against multidrug-resistant (MDR) strains [113].

When considering synergistic effects, a key finding is that clinical studies on polyphenols are becoming more important due to their proven ability to work together with traditional antibiotics. This helps make the antibiotics more effective against bacteria [114]. Polyphenols, even at subinhibitory concentrations, can enhance the action of an antibiotic against a bacterium that was initially resistant. For example, kaempferol and quercetin, both flavonols with intrinsic antimicrobial activity, increased the efficacy of rifampicin against rifampicin-resistant MRSA strains by 57.8% and 75.8%, respectively [115,116]. Flavanols like epicatechin gallate (ECg) and (−)-Epigallocatechin gallate (EGCg) enhance the effectiveness of antibiotics by weakening bacterial defenses. This process works in a few key ways. For example, some of these compounds can inhibit topoisomerases, a type of enzyme crucial for bacterial DNA synthesis, and show a synergistic effect with antibiotics like ciprofloxacin.

Additionally, ECg can make MRSA strains more susceptible to β-lactam antibiotics, such as penicillin or oxacillin. It does this by binding to the bacterial cell membrane, which disrupts a protein called PBP2a that is responsible for antibiotic resistance. ECg can also reduce the formation of biofilms and the production of virulence factors. Similarly, EGCg improves the effectiveness of antibiotics that target the bacterial cell wall and can also interfere with virulence factors like penicillinase [117].

A compelling example of synergy between polyphenols and antibiotics involves the combination of catechin and epicatechin gallate, extracted from Fructus crataegi, with antibiotics typically ineffective against MRSA, such as ampicillin [118], ampicillin/sulbactam [119], cefazolin [120], cefepime [121], and imipenem/cilastatin [122,123]. These combinations proved effective against MRSA in both in vitro and in vivo mouse infection models. The enhanced effect was largely attributed to the polyphenols’ ability to inhibit efflux pump genes, leading to the accumulation of antibiotics inside the bacterial cell [123]. Beyond antibiotic combinations, polyphenols also exhibit synergy among themselves, as observed with EGCg and quercetin against MRSA, a synergy attributed to a co-permeabilization process that facilitates compound entry into the cell [124]. Synergistic activity has also been documented between quercetin-3-glucoside, punicalagin, ellagic acid, and myricetin in various proportions and combinations against *S. aureus* CECT 59 [125,126].

The use of complex plant extracts, derived from various plant parts, is a common and effective approach in traditional medicine. These extracts exhibit diverse compositions, which can be further modulated by varying extraction conditions such as time, temperature, solvents, and pressure [127]. Research consistently shows that extracts from plants have antimicrobial properties and that their active compounds work together to fight off bacteria. For example, extracts from Lantana camara leaves are particularly effective against a range of clinical bacterial strains, including MRSA, *Streptococcus pyogenes*, VRE, and several others. This demonstrates how plant-based compounds can be a potent tool in the fight against various pathogens [128]. Ethanolic extracts of *Anthocephalus cadamba*, *Pterocarpus santalinus*, and *Butea monosperma* Lam. have also shown antimicrobial activity against MDR clinical isolates of 10 different microbial species, including *S. aureus*, *Acinetobacter* sp., *E. coli*, and *P. aeruginosa* [129]. The multifactorial and multi-target nature of the compounds within plant extracts can significantly impede the development of bacterial resistance [129].

In synthesis, the broad synergistic potential of plant-derived compounds, particularly polyphenols, stems from their ability to attack multiple resistance mechanisms simultaneously—such as inhibiting efflux pumps and disrupting bacterial membranes—which effectively resensitizes resistant pathogens like MRSA to conventional antibiotics, most notably β-lactams [129].

Table 1 provides a concise summary of the natural products discussed in this review, detailing their producing organism, type, target, mechanism of action, and main use.

Natural plant compounds have great chemical variety, but their use is often complicated by their complex makeup and limited supply. To overcome this, it is essential to progress from using simple plant extracts to isolating and creating specific, active molecules [135]. This change is crucial for meeting the strict requirements of today’s pharmacology, such as consistent dosage and toxicity testing, which are necessary for developing safe and effective treatments [135].

## 3. Mechanism of Action

**Figure 4:**
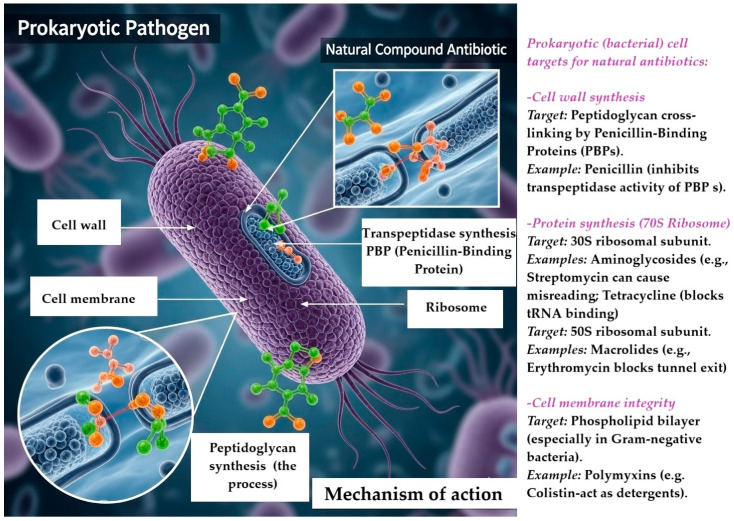
Mechanisms of action of natural antibiotics targeting prokaryotic pathogens.

Natural antimicrobials operate through a range of intricate mechanisms, often targeting essential processes within bacteria [136]. Figure 4 illustrates the action mechanism of natural antibiotics against prokaryotic pathogens, featuring a depiction of a prokaryotic bacterial cell with highlighted crucial antibiotic targets. The primary targets for these antibiotics include the cell wall—specifically the peptidoglycan layer and penicillin-binding proteins (PBPs)—as well as the 70S ribosome.

These various strategies can be categorized into several key groups.

Certain compounds, such as AMPs, melittin, and teixobactin, primarily function by directly assaulting the bacterial membrane. They do this by creating pores or destabilizing the lipid bilayer, leading to bacterial cell death. This mechanism of action is similarly observed in various polyphenols, such as epigallocatechin gallate, punicalagin, and quercetin [137,138]. Another significant target is the cell wall, where agents such as vancomycin, amphomycin, dalbavancin, ramoplanin, teixobactin, cephalosporin, cephamycin C, fosfomycin, moenomycin, penicillins, thienamycin, and teicoplanin disrupt peptidoglycan synthesis or compromise the structural integrity of the bacterial cell wall [139,140].

Interfering with protein synthesis represents a well-documented and efficient antimicrobial strategy. A diverse array of compounds—including albomycin, apramycin, chloramphenicol, and tetracycline—employ this approach by specifically targeting and disrupting different ribosomal functions or other cellular processes critical for protein production in microbes [141,142].

Another important antimicrobial mechanism focuses on bacterial DNA and RNA. Compounds like clorobiocin, coumermycin, kibdelomycin, daptomycin, rifamycin, and lipiarmycin directly interfere with bacterial replication or transcription processes [143,144].

Natural products demonstrate diverse antimicrobial mechanisms that extend beyond direct targeting of vital bacterial elements. For instance, baulamycin interferes with siderophore biosynthesis, essential for iron acquisition in bacteria [145]. Mirandamycin disrupts sugar metabolism and quinoloxidase activity in bacteria while reducing reactive oxygen species (ROS) production [16]. Additionally, pyridomycin inhibits mycolic acid synthesis—a crucial component of mycobacterial cell walls. These instances illustrate how natural compounds can effectively combat pathogens through various means that provide an edge over traditional drugs often limited to single-target methods [146]. A noteworthy area where natural antimicrobials excel is biofilm disruption—especially relevant for chronic infections. Compounds like skyllamycins, resveratrol, alginate lyase, ursolic acid, zingerone, cranberry proanthocyanidins, casbane diterpene, manoalide, solenopsin A, catechin, naringenin, ajoene, rosmarinic acid, eugenol, bergamottin, emodin, and baicalein can either hinder biofilm formation or actively dismantle existing ones [147]. Furthermore, efflux pump inhibition (EPI) serves as an important mechanism whereby various polyphenols (e.g., baicalein, capsaicin, indirubin, kaempferol rhamnoside, olympicin A, sarothrin, cumin, catechol, catharanthine, gallotannins, 4-hydroxytetralone, ursolic acid, lysergol, berberine, palmatine, EGCg, resveratrol, and pinosylvin) help restore effective intracellular concentrations of antibiotics by blocking the efflux pumps responsible for expelling antimicrobial agents from bacteria [123,148].

Resistance to natural antimicrobials tends to arise less frequently compared to conventional synthetic antibiotics due to the distinctive characteristics of natural compounds. Many natural products exhibit multi-target effects—disrupting multiple bacterial processes at once—which creates significant evolutionary challenges for bacteria trying to develop resistance mechanisms simultaneously [149]. This presents metabolic difficulties for bacteria and reduces their likelihood of developing resistance [144]. Additionally, many natural products interact with critical conserved pathways within bacteria. Mutations in these fundamental pathways typically lead to considerable fitness costs for bacterial survival when antimicrobial pressure is removed [150], deterring mutations associated with high fitness costs from persisting within populations [151]. Moreover, many natural antimicrobial target macromolecular structures like membrane or cell walls that are inherently hard for bacteria to modify without compromising viability [152].

Despite the inherent advantages offered by natural antimicrobials, some resistance has emerged over time. A prominent example is vancomycin, which remained effective for about thirty years until resistant strains of *S. aureus* were first identified [153]. This highlights that, even though natural products may exhibit prolonged efficacy durations, they are not entirely free from evolutionary pressures inducing resistance [154,155]. One notable resistance mechanism involves substituting D-Ala-D-lactate at the dipeptide termini during nascent peptidoglycan formation instead of typical D-Ala-D-Ala. This diminishes vancomycin’s binding capacity, significantly impacting its ability to interfere with cell wall construction [156]. Other resistant strains develop thickened walls full of free D-Ala-D-Ala ends, permitting them to sequester vancomycin, and thereby averting its action towards designated targets [156]. Further complexities surrounding vancomycin resistance include Atl amidase inhibition, which directs research towards creating new derivatives, exhibiting reduced affinity toward Atl and aiming to improve their effectiveness against MRSA [157].

While teixobactin shows no discernible resistance within its producing strain—and theoretically poses challenges toward developing resistance due its conserved target [84]—the dynamic nature surrounding bacterial evolution indicates that no antimicrobial remains indefinitely “resistance-proof”. Recent studies have revealed that certain enteric bacteria acquire resistances against plant-derived compounds, although their specific mechanisms remain poorly defined [148]. Environmental selection pressures appear influential—as evidenced by geckos exposed to medicinal plants, which exhibited diminished susceptibility among their resident strains towards the extracts derived from those plants [158]. Bacteria can adapt against complex naturals especially after extended exposure, making comprehensive insights into evasion tactics essential when designing durable therapies, encompassing alterations such as drug modification/inactivation, target alteration, and reduction in permeabilities, and changes in biofilm formations—all of which are achievable via mutations or horizontal gene transfers [5]. Continual vigilance alongside ongoing research remains imperative, even for those seemingly robust novel antibacterials; we must direct our attention towards next-gen derivatives/combinational regimens, circumventing emergent resistances [159].

### Synergistic Strategies

The strategic integration between conventional antibiotics along with natural compounds emerges as an exceptionally promising tactic amidst combating rising antimicrobial resistance. Such synergistic methodologies yield numerous clinical/developmental benefits, leveraging the unique attributes found within naturally derived substances, enhancing antibiotic effectiveness whilst potentially lowering dosage requirements, minimizing side effects, and refining pharmacokinetic (PK)/pharmacodynamic (PD) profiles [160].

Natural products elevate antibiotic functionality via several fundamental mechanisms. A pronounced advantage concerns membrane permeabilization, where polyphenols/natural agents destabilize cytoplasmic membranes, facilitating antibiotic ingress into previously protected cells that are otherwise barred from entry [152,161]. Efflux pumps serve critical roles regarding AMR [162], expelling drugs and thus compromising intracellular concentrations/making them less efficacious. Natural constituents, particularly phytochemicals, inhibit these efflux systems, reinstating effective levels inside cells reversing established resistances and thereby revitalizing the older antibiotics that were rendered ineffective due widespread efflux-mediated barriers [163,164].

Compelling evidence indicates that phytochemicals can effectively inhibit various efflux pumps in both Gram-positive and Gram-negative pathogenic bacteria. For instance, baicalein, capsaicin, indirubin, kaempferol rhamnoside, olympicin A, and sarothrin have been shown to successfully inhibit the NorA efflux pump in *S. aureus* [123]. Additionally, cumin has been found to modulate resistance against MRSA by targeting the LmrS efflux pump [165]. Plant-derived compounds such as 10-S-10-acetoxyeugenol acetate, catechol, catharanthine, and gallotannins have demonstrated an ability to block the ethidium bromide efflux pump (EtBr) in Mycobacterium smegmatis, *P. aeruginosa*, and uropathogenic *E. coli* [123]. The Yojl efflux pump in multidrug-resistant (MDR) *E. coli* is inhibited by 4-hydroxy-tetralone, ursolic acid and its derivatives, and lysergol [166]. Both berberine and palmatine are known to inhibit MexAB-OprM in clinical isolates of MDR *P. aeruginosa* [148]. Whole-plant extracts also function as efflux pump inhibitors (EPIs), showing synergistic effects when combined with conventional antibiotics. For example, phytochemicals present in Rhus coriaria seed extract significantly enhance the effectiveness of multiple antibiotics—such as oxytetracycline, penicillin G, and enrofloxacin—against multidrug-resistant *P. aeruginosa*, primarily by inhibiting efflux pumps [167]. A particular catechin known as (−)-epigallocatechin gallate (EGCg) sensitizes *S. aureus* to tetracycline through the inhibition of the Tet (K) efflux pump, thus increasing intracellular antibiotic retention [168]. Stilbenes like resveratrol and pinosylvin also serve as EPIs against antibiotic-resistant *Arcobacter butzleri*, sometimes fully reversing resistance [169].

In addition to their role in inhibiting efflux pumps, certain polyphenols such as catechins have been shown to interact directly with the lipid bilayer of bacterial membranes [161]. This interaction leads to thermotropic changes that increase membrane permeability and contribute to antimicrobial effects [170]. Lipophilic hydrocarbons found in plant extracts can destabilize cytoplasmic membrane structures while interacting with hydrophobic protein regions [171]. This mechanism elucidates how these compounds may enhance antibiotic efficacy by promoting uptake and interfering with resistance proteins. Specifically, (−)-epicatechin gallate (ECg) binds to the staphylococcal cell wall, causing biophysical alterations that disrupt β-lactam resistance machinery, and consequently restoring MRSA’s sensitivity to oxacillin and other β-lactam antibiotics [172].

Disruption of biofilms presents another effective synergistic strategy since infections associated with bacterial biofilms are notoriously challenging due to their reduced susceptibility to antibiotics [173]. A variety of natural compounds possess the capability to prevent biofilm formation or actively dismantle existing biofilms, thereby allowing for more effective antibiotic delivery within these structures—an important factor when addressing the persistent infections caused by biofilms from organisms like *Pseudomonas aeruginosa*.

Several diverse compounds exhibit anti-biofilm activity, including alginate lyase, ursolic acid, zingerone among others. Notable examples for targeting *Pseudomonas aeruginosa* biofilms include cranberry proanthocyanidins, casbane diterpene, manoalide, solenopsin A, catechin, naringenin, ajoene, rosmarinic acid eugenol bergamottin emodin, and baicalein [174].

For instance, certain cranberry proanthocyanidins were shown to enhance gentamicin effectiveness in an in vivo infection model using *Galleria mellonella* [175]. Some compounds that disrupt biofilms also exhibit intrinsic antimicrobial properties, further boosting overall treatment efficacy. The ability of natural products to dismantle biofilms serves as a force multiplier for antimicrobial action; this reactivates previously ineffective antibiotics by overcoming a significant structural barrier associated with resistance. This mechanism differs from direct bactericidal actions, which hold considerable promise for chronic or recurrent infections [158].

General plant extracts additionally demonstrate synergistic antimicrobial effects against AMR bacteria. Extracts from Duabanga grandiflora can restore MRSA’s sensitivity to ampicillin via suppression of *mecA* gene expression responsible for producing PBP2a resistance protein [176]. Extracts derived from Acacia nilotica, Syzygium aromaticum, and *Cinnamum zeylanicum* exhibit antimicrobial potential against various AMR clinical isolates along with ATCC strains; *A. nilotica* shows minimum inhibitory concentration (MIC) value, reaching as low as 9.75 µg/mL against *K. pneumoniae* ATCC-700803, *Salmonella typhimurium* ATCC-13311, and *E. faecalis* ATCC-29212 [177]. Additionally, extracts from *Salvia* spp. and *Matricaria recutita* reveal strong synergy when paired with oxacillin [178]. The multi-target characteristics inherent within plant extract compounds inherently hinder bacterial resistance development. The “molecular promiscuity” exhibited by polyphenols alongside their capacity for multiple target engagement positions them favorably within network pharmacology research that is aiming to decipher their intricate interactions while facilitating design innovations for combination therapies [179]. The results stemming from combination studies involving plant extracts, paired with clinical antibiotics, consistently reveal synergistic enhancements; these findings are crucially important amidst efforts to combat AMR pathogens. Although developing novel synthetic antibiotics remains vital, sensitization achieved through phytochemicals proves equally essential in the pursuit of establishing effective, long-lasting treatments [180].

Utilizing established molecules that have already been utilized in relevant clinical evaluations (antibiotics) along with combined innocuous natural substances streamlines research and development processes towards the discovery of potential new therapies. This strategy offers a sustainable approach for addressing AMR challenges through repurposing current antibacterial agents. Combining these naturally enriched medications allows their safety profiles to be leveraged, in addition to establishing manufacturing methods; accordingly, we can speed up clinical translation while minimizing the economic burdens associated drug development [14,181].

Integrating natural products alongside traditional antibiotic regimens presents a pragmatic solution that can be used in the aim of restoring lost efficacies [102]. Nonetheless, it becomes imperative to extend beyond mere observation, focusing on the precise molecular interactions involved [182]. An optimal strategy lies in discerning the means by which these combinations reduce the probabilities that arise from resistant formations—this aspect is central to the evaluation of long-term usefulness.

## 4. Incorporation of Natural Antibiotics in Drug-Delivery Systems

The clinical applications of many natural antibiotics are hindered by their inherent limitations, such as their poor stability, low bioavailability, and non-specific targeting. Advanced drug-delivery systems offer a promising strategy for overcoming these challenges by improving their pharmacokinetic profiles and enhancing their localized efficacy. In addition to the problems posed by the limited supply of antibiotics, many of the current antibiotics in use are losing effectiveness due to several issues, including harmful side effects on non-target areas, poor drug penetration at infection sites, lack of specificity, unpredictable or rapid release rates, and a continuous rise in the development of antibiotic resistance [183,184].

Unlike conventional antibiotics, natural bioactive compounds, that represent unmodified secondary metabolites derived from plants, fungi, microbes, and animals, offer a promising alternative for curing a wide range of diseases [185]. These naturally occurring substances, despite having the potential to overcome many of the restrictions related to traditional antibiotics, also tend to exhibit better compatibility with drug-delivery systems. In this way, local antibiotic delivery has emerged as a very prospective potential strategy to address the challenges and limitations of systemic antibiotic treatment. For example, in compromised wounds, especially those with avascular regions, blood flow could be insufficient to transport antibiotics to the site of infection [186]. Systemic administration can result in adverse side effects such as toxicity throughout the body. On the other hand, localized drug-delivery systems facilitate high concentrations of antibiotics being delivered directly to the infection site, maintaining the overall serum levels with low systemic risks [187]. As a result, the ideal local delivery system should maximize the concentration of antibiotics where they are needed, with minimal potential for systemic toxicity [186]. It is believed that there is still an enormous number of undiscovered bioactive molecules, especially from plants, which remain to be explored [185].

As a result, it is crucial to develop more efficient and effective drug-delivery systems for improving the existing traditional methods or techniques such as oral or intravenous administration, particularly in the fight against bacterial infections. Antibiotics must be delivered at concentrations that exceed the minimum inhibitory concentrations (MICs) and to eliminate existing infections which surpass the minimum bactericidal concentration (MBC) in order to prevent infections [188]. The benefits of localized and controlled antibiotics release include lower overall dosing, minimized toxicity, extended duration of the drug or antibiotic activity, and limited systemic exposure. It is possible to deliver high doses specific to the bacterial strain involved without exceeding systemic toxicity thresholds by focusing the antibiotic directly on the specific infection site. In this way, it is possible to reduce the occurrence of side effects and prevent the development of AMR [189]. By avoiding systemic antibiotic administration, it is possible to enhance patient adherence to treatment. Thus, controlled and sustained release could offer a clear way to achieve this goal by enhancing PK and PD profiles [190].

The Effect of Carrier Matrices on Nanoparticle Antimicrobial Activity

The carrier matrix in which nanoparticles are embedded significantly influences their antimicrobial activity by controlling the release of the active agents and, in some cases, by possessing their own inherent antimicrobial properties [191]. This is a critical aspect of designing effective antimicrobial materials, especially for applications like medical devices [192], wound dressings [193], and food packaging [194].

A major effect of the carrier matrix is its ability to modulate the release kinetics of the active antimicrobial compounds [194]. Without a suitable matrix, nanoparticles can exhibit a “burst release”, where a large amount of an antimicrobial agent is released immediately upon contact with the environment [195]. While this can provide an initially potent effect, it can quickly deplete the active agent, leading to a short-lived antimicrobial effect and potentially causing local toxicity. The carrier matrix provides a scaffold that can control the rate and duration of the release. This sustained release mechanism is achieved through various interactions between the nanoparticles, the active compound, and the matrix itself [195]. For example, in a polymer or mesoporous silica matrix, the active compound can be physically encapsulated or chemically bonded within the pores or polymer chains [196]. The release rate is then governed by factors such as the following: diffusion, i.e., the rate at which the active compound diffuses out of the matrix pores or through the polymer network; matrix degradation, i.e., the gradual breakdown or dissolution of the carrier material, which slowly frees the embedded nanoparticles or antimicrobial agents [197]; finally, the release can be triggered or accelerated by specific environmental conditions like changes in pH or temperature, which cause the matrix to swell or degrade [198]. For example, a polymer matrix can be designed to swell and form a gel layer upon contact with an aqueous environment, such as a wound [197]. This gel layer acts as a barrier, slowing down the diffusion of the antimicrobial agent and preventing burst release [197]. A mesoporous silica matrix, with its well-defined and tunable pore sizes, can physically trap the antimicrobial agents, releasing them slowly as the surrounding fluid diffuses into the pores. This mechanism ensures that a consistent, effective concentration of the antimicrobial agent is maintained over a longer period, preventing the growth of bacteria and reducing the risk of resistance [199].

In addition to their role in release kinetics, some carrier matrices, like certain polymers and mesoporous silica, can possess their own intrinsic antimicrobial properties [199]. This dual functionality creates a synergistic effect that enhances the overall antimicrobial performance [200]. Some polymers, like chitosan, are naturally antimicrobial. Chitosan’s positive charge allows it to interact with the negatively charged cell membranes of bacteria, disrupting them and leading to cell death [201]. When nanoparticles (e.g., silver nanoparticles) are embedded in a chitosan matrix, the combined effect can be more potent than either component alone [202]. The chitosan provides immediate contact killing, while the silver nanoparticles offer a long-term, sustained effect. The surface chemistry of mesoporous silica can be modified to be antimicrobial. For instance, by functionalizing the silica with quaternary ammonium groups, the surface becomes positively charged, enabling it to kill bacteria on contact. This provides an additional line of defense beyond the embedded antimicrobial agents [203]. The high surface area and porous structure also make it a suitable platform for delivering reactive oxygen species or metal ions (e.g., silver ions) that can damage bacterial cells. The choice of carrier matrix is therefore not just a matter of structural support but is a strategic decision that can significantly impact the efficacy and longevity of an antimicrobial material. It allows for the design of systems that provide both immediate and sustained antimicrobial action, which is vital for preventing and treating infections [203]. Recent advancements in bioengineering have developed improved drug-delivery systems that are capable of overcoming the current limitations and sustaining the release of natural antimicrobial agents such as nanoparticles [204], hydrogels [205], liposomes [206], solid lipid nanoparticles [207], stimuli-responsive platforms [183], and so on. In this section, we summarize the latest developments in drug-delivery systems, including the advancements mentioned, incorporating several natural antibiotics with different local applications.

### 4.1. Nanoparticles

Nanotechnology represents a science which determines the preparation of particles with dimensions ranging from 1 to 1000 nm [208] using a variety of synthesis methods, along with techniques to modify their structure and size. Nanoparticles (NPs) find their applications in several domains such as molecular biology, physics, organic and inorganic chemistry, materials science, and so on [208]. Recent advancements in nanotechnology have made a substantial contribution to medicine and healthcare, especially for drug-delivery systems, as they are one of the most promising and progressive applications in this domain [187,208]. These nanoparticle-based delivery systems can evade immune detection, control release profiles, improve antibiotic solubility, target drugs to specific sites, and even carry multiple therapeutic agents simultaneously [187,209,210,211]; in this way, NPs can deliver combination therapies through a single system. Antibacterial performance can be enhanced due to such strategies by leveraging synergistic effects and lowering the chances of resistance emergence [211].

Building on these points, Table 2 summarizes the key features and applications of various nanoparticle-based systems for natural antibiotic delivery, providing a clear overview of their advantages and the specific examples discussed in this section.

Due to these characteristics, both the PK and PD profiles are improved compared to their conventional counterparts [187]. Therefore, NPs have emerged as highly promising drug-delivery systems that are capable of overcoming several current medical limitations or challenges. Other characteristics, including enhanced bioavailability, reduced toxicity, and precise targeting, make NPs effective for therapeutic applications for drug-delivery systems. In addition to this role, NPs also exhibit strong antimicrobial activity, including antibacterial, antifungal, antiviral, and antiparasitic effects, including improved stability and potential for more accurate evaluation of therapeutic effects using different tools [209,218].

**Figure 5:**
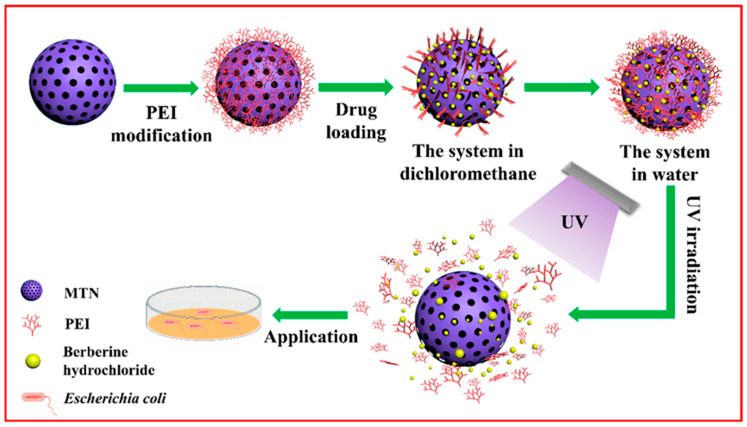
Schematic representation of the preparation process of BH-loaded MTN-PEI and its release of cargos in response to UV light [219].

As a result, targeted natural antibiotic delivery using nanoparticles represents a valuable strategy as it allows for controlled release directly at the infection site [218]. For instance, berberine and its derivates could be integrated into different types of nanoparticles for targeting specific sites. F. Zuo et al. [219] synthesized mesoporous titanium nanoparticles (MTN) using a sol-gel method coated with functionalized UV-responsive ethylene imine polymer (PEI) to encapsulate the natural antibiotic berberine hydrochloride (BH). They used PEI layer, which is UV-degradable, for enhanced release control of BH; this feature was utilized to analyze the synergistic antibacterial activity of BH and MTN against *Escherichia coli* (*E. coli*) and to demonstrate the UV-triggered antimicrobial capability of this system, BH-MTN-PEI (Figure 5). In this way, this system demonstrated a high drug-loading capacity of 22.71 ± 1.12% and an encapsulation efficiency of 46.56 ± 0.52%, which were attributed to the high specific surface area of MTN. The antibacterial evaluation revealed that BH-MTN-PEI exhibited UV-dependent release behavior with a bacterial lethality of 37.76% against *E. coli* after a short interval of only 8 min. Therefore, this novel system proposed by F. Zuo et al. [219] successfully demonstrated the efficient inhibition of *E.coli*, offering a promising approach for developing novel antimicrobial therapeutics [170].

Berberine and its derivates can be incorporated and absorbed by biodegradable natural polymer nanoparticles. Their biomechanical properties, low cytotoxicity, and biocompatibility make polymers suitable for the development of novel drug-delivery systems [220]. For example, a study involving the incorporation of berberine in polymeric nanoparticles was conducted by A. Gunor Ak et al. [221]. They developed a mode of incorporating berberine chloride (BER) into chitosan nanoparticles (BER-NPs) to enhance the oral bioavailability and gastrointestinal stability of BER. They prepared this system using an ionotropic gelation technique [221]. The characterization of the BER-NPs revealed promising results: the encapsulation efficiency had a value of 31.5 ± 0.4% and a zeta potential of 41.6 ± 0.8 mV [221]. Their physicochemical studies showed the improved solubility and partitioning behavior of BER-NPs compared to simple berberine. As a result, they fabricated BER-NPs with enhanced protection of berberine in gastrointestinal fluids and with a substantial increase in systemic absorption [221]. Another study, which covers the suitable incorporation of berberine in polymer nanoparticles, was carried out by S. Comincini et al. [222]. They prepared two berberine hydrophobic salts, dodecyl sulfate (S) and laurate (L), and encapsulated them into PLGA (poly lactic-co-glycolic acid)-based nanoparticles, with surface modification achieved by chitosan oleate coating [222]. Also, the NPs were functionalized with folic acid to enhance the targeted delivery function. These loaded NPs demonstrated notable efficacy in including cytotoxicity in glioblastoma (GBM) T98G cells [222]. Their results highlight their potential usage as mitochondria-targeting nanomedicine platforms, which are suitable for photodynamic therapy; these findings could represent a significant advancement toward effective therapeutic options for malignant gliomas [222].

Generally, NPs represent a promising solution for drug-delivery systems as they possess various advantages and less drawbacks [103]. The carrier matrix in which NPs are incorporated plays a crucial role in determining their safety profile, efficacy, and stability [102]. As a result, future studies should cover the therapeutic potential of NPs and could address the toxicity concerns surrounding NPs to validate both their safety and efficacy [103].

### 4.2. Hydrogels

Hydrogels represent biomaterials that are typically formed from water-soluble natural or synthetic polymers that undergo gelation in response to several stimuli, including temperature, ionic strength, pH, or ultraviolet exposure [205]. Hydrogels are generally soft 3D (three-dimensional) networks of linked polymers that are capable of supporting large water volumes within their structure [223]. These materials have different characteristics in size, architecture, and function, and the combination of these features makes hydrogels suitable for use in drug-delivery systems. They can be fabricated into almost any overall size and shape, dictating several of their properties. For example, the macroscopic design of hydrogels provides routes by which hydrogels can be targeted in the human body. On the other hand, the micropores present in hydrogels can affect their physical properties while also facilitating connective drug transport [212]. Moreover, hydrogels can be used as a versatile platform for the development of several therapies, allowing the delivery of multiple antibiotics or therapeutic agents within a single system to enhance their efficacy against infections [223]. Due to their hydrophilicity, unique 3D network structure, excellent biocompatibility, and capacity to support cell adhesion, hydrogels are considered to be highly suitable carriers for drug-delivery systems and localized antibacterial applications [205].

**Figure 6:**
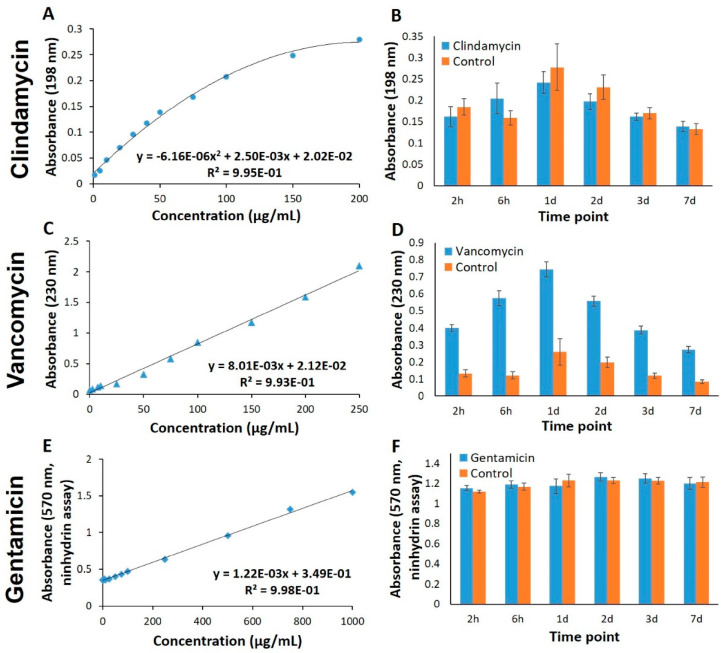
Spectrophotometric quantification of antibiotics. Standard curves (**A**,**C**,**E**) obtained by measuring the absorbance at 198 nm and 230 nm; Ninhydrin assay of defined concentrations of clindamycin, vancomycin, and gentamicin in 0.9% NaCl solution, respectively. Analysis of the release solutions (**B**,**D**,**F**) obtained from antibiotic-loaded and unloaded (control) ALG-MC scaffolds, using the respective method (n = 5) [214].

The incorporation of natural antibiotics into hydrogels could provide controlled, localized delivery and improved antimicrobial efficacy. For example, A.R. Akkieni et al. [214] developed an antibiotics-loaded hydrogel-delivery system, which was fabricated through a 3D core/shell extrusion-printing technique. They integrated natural antibiotics, such as vancomycin, clindamycin, or gentamicin, into this system, making it suitable for treating soft tissue infections. The core hydrogel is loaded with antibiotics, serving as a reservoir, while the shell hydrogel determines the degree of mechanical support and behaves like a diffusion barrier [214]. Alginate was chosen as the base material due to its excellent biocompatibility and its vast use in tissue engineering and clinical applications. The printability of the material has been improved by blending alginate (ALG) with methylcellulose (MC); this combination is compatible with various cell types. Further modifications included adding laponite (LAP), enhancing the material properties of the system [214]. The release profiles of the antibiotics through the shell were examined along with the absorbance of pure antibiotic solutions at specific excitation wavelengths (Figure 6). Thus, the results show that the alginate shell component is a key factor in determining the mechanical stability of the scaffolds and can influence the release kinetics of loaded antibiotics [214]. Moreover, incorporating LAP into the ALG-MC shell hydrogel system was found to slow down the release kinetics; meanwhile, adjusting the shell thickness enables the additional modulation of the release of natural antibiotics [214].

R.B. Mbuku et al. [216] evaluated the effectiveness of a thermosensitive hydrogel formulation fabricated of poloxamer P407, incorporating a tri-enzymatic combination of DNA/RNA endonuclease, endo-1,4-β-D-glucanase, and β-N-acetyhexosaminidase alongside a super-therapeutic-concentration dose of vancomycin for the localized treatment of biofilm-related infections [216]. In vitro studies have demonstrated that the hydrogel formulation enabled the controlled release of the active components over a 12 h period. A single local dose of the hydrogel, incorporated with vancomycin and the tri-enzymatic combination, was more effective compared to a systemic-only hydrogel or systemic vancomycin alone [216]. This effective combination has been demonstrated to have significant potential for treating biofilm-associated infections, targeting *Staphylococcus aureus* (*S. aureus*) on implantable medical devices due to the hydrogel’s ability to achieve the localized delivery of high antibiotic concentrations in combination with the enzymatic disruption of the extracellular matrix, enhancing antimicrobial penetration [216]. Also, F. Jiang et al. [224] developed a dual-crosslinked and dynamically crosslinked hydrogel consisting of acid-grafted oxidized hyaluronic acid (OHA-PBA), which encapsulates gentamicin (Gen), used as a dynamic crosslinking agent, and PVA (Polyvinyl alcohol), that participates in the boronic acid ester bonding to improve the hydrogel’s structural injectability, integrity, and self-healing properties. This system (OHA-PBA/PVA/Gen), which is nontoxic to mammalian cells, provided strong antimicrobial activity against both *S. aureus* and Gram-negative bacteria *E. coli* through contact and diffusion killing mechanisms. This unique combination of dual-crosslinked hydrogels with gentamicin has potential to be used as a versatile dressing for treating bacterial infections [224].

Overall, hydrogels represent a suitable alternative option for use as carriers for natural antibiotics due to their 3D network structures, which enable the incorporation of multiple therapeutic agents and supports tissue regeneration [225]. However, their current challenges and limitations could potentially be addressed through continuous research for highlighting their potential as versatile platforms for natural antibiotic delivery [102].

### 4.3. Liposomes

Liposomes are small, spherical vesicles composed of a minimum of one phospholipid bilayer with an inner aqueous compartment or unit [226,227,228]. Their structure includes a polar hydrophilic head, which determines the surface properties of liposomes, and a nonpolar hydrophobic tail, which provides the fluidity of the membrane of liposomes [228]. The spherical shell structure with an aqueous core that liposomes possess is capable of encapsulating various bioactive compounds, such as proteins, hormones, and antibiotics [229]. This unique architecture means that liposomes are one of the most used, studied, and promising nano-vehicles for carrying and delivering antimicrobial agents, such as natural antibiotics [229,230]. They present a series of advantages, such as excellent biocompatibility, and are completely biodegradable, nontoxic, and non-immunogenic. Liposomes are capable of encapsulating, delivering, and sustaining both hydrophobic and hydrophilic drugs. They can change the biodistribution of the drug, coupling with specific ligands and modulating the PK and PDs of encapsulated agents [229]. As a result, liposomes are considered attractive carriers for antibiotics delivery, being the most well-developed and -established drug-delivery systems available among various types of nanoparticles [226,229].

In this way, liposomes are widely used for the delivery and release of natural antibiotics. For instance, a recent study by N. M. Alzahrani et al. [231] investigated a new strategy for combating *Pseudomonas aeruginosa* biofilms in cystic fibrosis patients. They enhanced the antimicrobial efficacy of the natural-product-derived antibiotic tobramycin by loading it into liposomal nanoparticles in combination with an anti-biofilm peptide (IDR-1018). While this delivery method did not alter the direct antibacterial activity of tobramycin, the overall system is a promising approach for improving therapeutic outcomes and reducing the required dose for treating lung infections.

Vancomycin is a natural antibiotic that can be incorporated in liposomes as well as in hydrogels systems. M. Vatankhah et al. [232] researched multivesicular liposomes (MVLs), developing a controlled-release delivery system as a carrier for the local delivery of vancomycin hydrochloride (VAN HL). M. Vatankhah et al. utilized a double-emulsion technique to create multivesicular liposomes (MVLs), which were then loaded with a natural antibiotic using an ammonium gradient method. An analysis of their antibacterial effectiveness via a disc diffusion assay revealed that the optimized MVLs achieved a drug encapsulation efficiency exceeding 90%.

**Figure 7:**
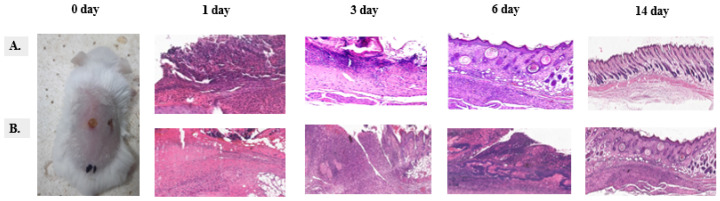
Histological evaluation of skin tissue following treatment with liposome-encapsulated vancomycin and free vancomycin. Staining: H&E, Magnification ×20. Abbreviations: The 0-day image shows the condition of the skin tissue after a 0.5 mm biopsy punch was used to create a wound in both groups before starting the experiment. Skin histological analyses were performed on days 1, 3, 6, and 14 of the experiment. (**A**) Liposome-encapsulated vancomycin group; (**B**) free vancomycin group [233].

The study’s kinetic analysis demonstrated a significant difference in drug release profiles: while free vancomycin was released within 6–8 h, passively loaded liposomes extended the release to approximately 6 days, and the actively loaded MVLs provided sustained drug delivery for up to 19 days. These findings indicate that the MVL system developed by M. Vatankhah et al. offers optimal particle characteristics and favorable release kinetics, underscoring its potential for targeted antibiotic delivery in the treatment of osteomyelitis. In another similar work, E. Erdene et al. [233] evaluated the efficacy of the incorporation of vancomycin against MRSA. This achieved complete bacterial inhibition against MRSA, outperforming the free form of natural antibiotics. In vivo experiments have been performed involving a mouse wound infection model, where full-tissue regeneration was observed by the 14th day. A comparative histological evaluation of skin tissue following treatments with liposome-encapsulated VAN and free VAN is presented in Figure 7. This histological analysis confirmed a reduced inflammatory response and improved tissue repair in the liposome-treated group, while the free vancomycin exhibited ongoing necrosis and fibrosis [233].

Overall, the liposomal system provided prolonged drug release and enhanced PKs, suggesting a lower risk of resistance development, minimized side effects, and improving clinical outcomes as a promising scalable therapeutic strategy against resistant bacteria such as MRSA. This highlights the fact that liposomes are effective carriers for use in targeted antibiotic delivery systems, ensuring safe and efficacious treatment [233].

Liposomes have proven to be an exceptional and adaptable method for administering natural antibiotics. Their distinct architecture, featuring a lipid bilayer surrounding a watery core, allows them to carry both water-soluble and fat-soluble drugs [233]. This capability helps overcome common issues with conventional antibiotics, like their low bioavailability and how quickly they are cleared from the body [102]. Research on encapsulating natural antibiotics, such as tobramycin and vancomycin, highlights the considerable advantages of this technique. Specifically, liposomal formulations extend the drug’s release time, boost its effectiveness, and lead to better patient outcomes by allowing for a lower required dose and fewer side effects [102]. The remarkable success of liposome-based vancomycin in treating MRSA infections—seen through better tissue healing and reduced inflammation—further emphasizes their potential [233]. Ultimately, liposomes are a viable and promising solution for fighting antibiotic-resistant bacteria by providing a targeted and consistent way to deliver medication, thereby making treatments safer and more effective.

### 4.4. Solid Lipid Nanoparticles (SLNs)

Lipids have often been used as drug-delivery systems and excipients for enhancing the solubility of lipophilic drugs with low water solubility [234]. Among these various lipid-based nanocarriers, solid lipid nanoparticles (SLNs) represent a solid and promising option for traditional colloidal systems such as liposomes, lipid emulsions, and polymeric nanoparticles [235,236]. First developed by Muller and Lucks in 1996 [234] as tiny and spherical particles fabricated by solid lipids at ambient temperature, they still represent versatile carriers as stable, nontoxic, and reliable particles in drug delivery [234,237]. A primary advantage of SLNs is their low toxicity, a feature attributed to their composition of physiological lipids. In addition to this key safety profile, SLNs offer numerous other benefits, including high stability for encapsulated drugs and the ability to accommodate both hydrophilic and lipophilic compounds. These nanocarriers are also easy to sterilize, can be produced on a large scale, and support lyophilization for long-term storage, while enabling targeted and controlled drug release [235,236]. Thus, drug release from SLNs is influenced by matrix composition and the positioning of the drug within the formulation. SLNs have proven to be effective drug-delivery systems and could define the future of lipid-based delivery based on their unique and valuable characteristics [235].

**Figure 8:**
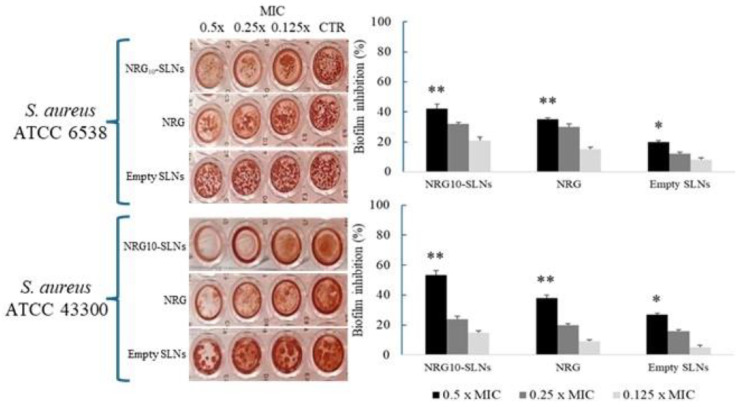
Effects of different concentrations (from 0.5× MIC to 0.125× MIC) of NRG_10_-SLNs, free NRG, and empty SLNs on biofilm formation in *S. aureus* ATCC 6538 and *S. aureus* ATCC 43300 (MRSA). Data are presented as mean ± SD, * *p* < 0.05, ** *p* < 0.01 vs. control [238].

In a study conducted by F. de Gaetano et al. [238], solid lipid nanoparticles were loaded with flavanone naringenin (NRG) for treating several bacterial infections. The NRG-SLNs were prepared using a solvent emulsification/diffusion and ultrasonication technique. A specific formulation, NRG_10_-SLNs (containing a 10% theoretical amount of NRG), demonstrated strong physical stability for over four weeks [238]. In vitro analyses of these NRG_10_-SLNs showed their antibacterial and antibiofilm properties, which were then compared to those of free naringenin [238]. Thus, the NRG_10_-SLNs demonstrated bacteriostatic effects against *S. aureus*, including MRSA. Results indicated that NRG_10_ was the most effective in inhibiting biofilm formation compared to free NRG. More specifically, in Figure 8, it is shown that biofilm reduction rates reached 42% for *S. aureus* ATC 6538 and 53% for ATTC 43000 (MRSA), whereas the corresponding reductions for the free NRG were 35% and 38%, respectively [238]. The research highlights the significant efficacy of NRG_10_-SLNs working as an excellent antibacterial system towards *S. aureus* strains, providing a foundation for the development of a liquid pharmaceutical preparation in the management of topical ocular infections [238].

M. H. Kharaji et al. [239] performed research into the effects of the oral administration of paromomycin sulphate (PM) loaded onto solid lipid nanoparticles (PM-SLNs) and their advantages over other colloidal carriers for treating Leishmania. In this study, the authors focused on quantifying the parasite load through real-time PCR, measuring the footpad swelling, and assessing the level of cytokines [239]. Overall, the PM-SLN oral formulation provided significant efficacy and safety in treating cutaneous Leishmaniasis by effective reduction in parasite burden and modulation of the immune response in infected mice used as a model. Also, the enhanced therapeutic outcome is explained by the improved macrophage uptake, leading to better drug accumulation at the infection site. This result confirms that the system of PM-SLNs represents an excellent approach as a drug-delivery system for the treatment of Leishmania [239].

Another study has focused on improving the targeted antibiotic therapy of vancomycin. R. S. Kalhapure et al. [240] developed pH-responsive solid lipid nanoparticles (SLNs) from a newly synthesized, acid-cleavable lipid (SA-3M) to enhance the targeted delivery of vancomycin to infection sites. The system, known as VM-FB-SA-3M-SLNs, achieved a notable 58% encapsulation efficiency and demonstrated faster vancomycin release at the acidic pH of infection (6.5) compared to the physiological pH (7.4). In vitro tests confirmed the improved activity of this formulation against both MSSA and MRSA in acidic conditions. Furthermore, in vivo studies using a mouse skin model showed an approximate 22-fold reduction in the MRSA bacterial load following SLN treatment compared to free vancomycin treatment [240]. Histological analysis also revealed minimal tissue inflammation, underscoring the potential of these SA-3M-based SLNs to be a precise, pH-responsive drug-delivery system for treating infections [240]. Thus, the incorporation of natural antibiotics into advanced and novel drug-delivery systems such as nanoparticles, liposomes, and solid lipid nanoparticles provides a promising strategy for enhancing antimicrobial efficacy, to enable the targeted and sustained release of antibiotics for treating several types of infection while reducing systemic toxicity and resistance development. These systems not only improve the PK and PD profiles but also provide enhanced therapeutic outcomes when they are formulated in such systems [240]. In Table 3, we present a systematic view of the most important advantages and limitations of these drug-delivery systems, incorporated with natural antibiotics. The choice of the carrier matrix is directly proportional with antimicrobial activity by controlling factors or parameters such as the release rate, toxicity, or target spots. By taking each system’s advantages and drawbacks into consideration, in addition to the environment in which natural antibiotics are incorporated as drug-delivery systems and their potential applications, it could be possible to determine which system is the most suitable. For example, NPs could be potentially chosen over other systems when controlled release and precise antimicrobial action is required. Liposomes are preferrable when it is crucial for the carrier to have good wettability and biocompatibility. When it comes to localized infections, hydrogels may be a better alternative for more sustained antimicrobial effects. Nevertheless, SLNs are considered to be a suitable option in challenging and corrosive environments where biocompatibility and drug stability are some key factors for effective therapy. Overall, the most suitable and appropriate drug-delivery system is bound to be variable and is strongly dependent on specific therapeutic needs.

Drug-delivery systems offer a practical approach for overcoming the PK challenges posed by natural compounds. However, the creation of these systems brings a set of hurdles, such as ensuring they are compatible with the body, that they can effectively target specific areas, and that they do not introduce new, unforeseen toxic effects [103]. The key difficulty lies in engineering systems that are not only efficient but which are also affordable and scalable for widespread clinical application.

## 5. Safety, Efficacy, and Toxicity of Natural Antibiotics

Natural antibiotics are known and used on a large scale, due to their excellent biocompatibility and their antimicrobial efficacy. Even though these antibiotics are natural, they are not exempt from concerns surrounding their safety, toxicity, or cytotoxicity. Nature-derived antibiotics, including benzylpenicillin, gentamicin, and cephalosporins, are usually associated with significant toxicity [244]. Other types of antibiotics, such as semi-synthetic antibiotics, such as ampicillin and amikacin [245], as well as fully synthetic antibiotics, including moxifloxacin [246], exhibit enhanced therapeutic efficacy while offering reduced toxicity compared to natural antibiotics [244].

A critical evaluation of natural antibiotics is essential if we are to thoroughly assess their safety, therapeutic efficacy, and potential for toxicity. To overcome these obstacles, continuous advances in drug-delivery systems, technologies, and structural modifications are imperative, allowing us to transform natural compounds and products into reliable and safe treatments. Table 4 summarizes the safety, efficacy, and key clinical considerations of selected natural antibiotics, highlighting their therapeutic potential alongside their inherent toxicity risks and optimization strategies.

While approximately 1–10% of the population reports a penicillin allergy, a notable portion of these individuals do not show evidence of a true IgE-mediated hypersensitivity upon formal testing. Despite this, clinicians frequently opt to avoid prescribing penicillin or other β-lactam antibiotics, even when these drugs may be the most suitable therapeutic choice for a patient [247]. Most reactions labeled as penicillin allergies are adverse effects that are not immunologically mediated hypersensitivity. Each time penicillin is administered to a patient with or without history of an allergy, there is a slight chance of their experiencing an adverse reaction (approximately 1–2%), and many of these reactions are inaccurately reported as new penicillin allergies. The frequency of newly reported penicillin allergies varies according to different factors, such as the patient’s sex, the nature of infection, and the timing of follow-up post-exposure. For example, the incidence of newly reported allergies is approximately 1.45% in females and 1.11% in males [248]. However, in their literature study, D. Vyles et al. [249] reported that penicillin allergy is over-diagnosed, especially during childhood, leading to unnecessary use of broad-spectrum antibiotics. Early de-labeling in children can reduce this lifelong burden and promote more appropriate antibiotic use. A validated three-tier testing method offers near 100% negative predictive value, but growing evidence supports the safety and economic advantage of direct oral antibiotic challenges in low-risk pediatric cases. Also, S. Fox et M. Park [247] describe the safety and efficacy of penicillin skin testing evaluation in the pediatric population. They demonstrated that penicillin skin testing is a reliable and safe diagnostic tool for evaluating potential allergies in children. The study, which involved over 700 pediatric patients, found no adverse reactions, confirming that this procedure can be used with minimal risk to the patient. Nevertheless, Ham et al. [250] performed a clinical study that evaluated a specific approach for assessing penicillin allergies, aiming to enhance antibiotic selection and patient outcomes. Adult inpatients with a recorded penicillin allergy were reviewed using a structured institutional protocol that prioritized direct oral two-step challenges over traditional skin testing. From the cohort assessed, 96% were safely de-labeled, with over half experiencing changes to more appropriate antibiotic therapy. Thus, these findings suggest the potential for pharmacist-driven protocols to safely remove inaccurate penicillin allergy labels.

Vancomycin, which is part of the Glycopeptides group of antibiotics, is often administered in hospitals, because of the increasing occurrence of sepsis and septic shock. Studies have proven that some specific cases are still inconclusive in terms of their safety and efficacy. A common limitation of vancomycin dosing is its lack of reliability in patients with acute kidney disease, as current guidelines are based on stable renal function. For patients with obesity, vancomycin’s PKs are altered by increased distribution volume and clearance. While dosing based on actual body weight is often recommended, this practice is not consistently supported by the existing PK evidence. Therefore, further research is needed to develop and validate more personalized dosing strategies for these specific patient populations [251]. G. Zhang et al. [252] performed a unique meta-analysis to highlight the efficacy and safety of vancomycin by comprehensively comparing vancomycin with a wide range of antibiotics used in the treatment of *S. aureus* bacteremia. It has been observed that a limitation was the inconsistent reporting of MICs, preventing the assessment of whether MIC creep contributed to vancomycin failure. Also, studies conducted especially in Europe and North America could lead to potential regional bias. Thus, vancomycin has been determined to be less effective and less safe in comparation with another natural antibiotic, daptomycin, in the management of *S. aureus* bacteremia; this is because vancomycin was linked to a considerable risk of adverse effects. However, vancomycin remains excellent in the treatment of *S. aureus* bacteremia, but is limited in comparison with other antibiotics. Another meta-analysis study has been conducted by H. Mei et al. [253], where they analyzed the clinical relevance of administering a vancomycin loading dose to assess the clinical efficacy and safety in the treatment of infections. It has been highlighted that the loading dose of vancomycin has been proven to significantly improve the potential of reaching therapeutic drug levels. This approach has been shown to have a reduced risk of nephrotoxicity, and it does not lead to the occurrence of other adverse effects. Overall, researchers have concluded that vancomycin loading doses represent a safe and effective strategy, especially for ill patients, but further study and research are necessary to fully establish its safety and efficacy.

Another key natural antibiotic used in the treatment of different infections is gentamicin. Gentamicin is part of the aminoglycoside group and it is mainly used in short-term empirical combination regimens. It is a bactericidal aminoglycoside that is effective against Gram-negative bacilli and it exhibits synergistic effects against β-lactams antibiotics [254]. Even though it has been used for over 50 years, its dosing remains complex and limited in specific groups of patients, such as the following groups: critically ill people, elderly people, and neonatal patients, for whom PK often derivates from typical adult parameters. C. J. Hodiamont et al. [255] performed a comprehensive review in which they summarized the PKs of gentamicin in different patient populations and examined the implications for optimal dosing in the treatment of Gram-negative bacterial infections, concentrating on developments made in the past decade. Research has shown that administering an initial dose of a drug based on total body weight is the most effective strategy for optimizing its therapeutic efficacy in both adults and children over one month. However, increasing this starting dose does not necessarily improve clinical outcomes and may instead increase the risk of kidney toxicity. Therefore, the use of therapeutic drug monitoring is recommended in mitigating this risk. Despite recent advances in population-level PK modeling for various patient groups, the precise efficacy targets remain unclear, especially in cases where gentamicin is the primary treatment. R. Ghoneim et al. [254] conducted an analysis using medical records to evaluate the population PKs of gentamicin patients aged 1 month–12 years. Using Monte Carlo simulation, the results showed the best data was described by two-compartment model, where a favorable probability of achieving PD targets is indicated even at a MIC dose of 2 mg/L. Thus, a strategy consisting of a single daily dose of gentamicin appears to be effective in pediatric patients, determining adequate target attainment while keeping gentamicin concentrations below the threshold associated with toxicity throughout. E. Best et al. [256] analyzed the safety of one dose of gentamicin per day in children and the necessity of therapeutic drug monitoring in a pediatric cohort. They monitored a total of 79 children who received 106 treatment episodes; it was observed that irreversible ototoxicity associated with once-daily dosing of gentamicin is relatively common but is limited to children with pre-existing conditions, and nephrotoxicity was rare and reversible. Regarding the toxicity profile of gentamicin, once-daily dosing could be favored for low-risk pediatric patients compared to traditional multiple doses per day. Therapeutic drug monitoring did not prove to be efficient in preventing toxicity and it has been determined that it may not be necessary for healthy children receiving short-term treatment. In their case report, O. Kalmanson et al. [257] investigated gentamicin ototoxicity risk and identified suitable alternative antibiotics for penicillin-allergic patients who were undergoing surgery. In general, gentamicin is recommended when a patient suffers from a true penicillin allergy and cannot be treated with cephalosporins. Due to the risk of otologic toxicity, clinicians are advised to consider alternative antibiotics such as cefoxitin, ertapenem, or cefotetan while reserving gentamicin for situations where safer options are not feasible. Overall, gentamicin, similarly to aminoglycosides in general, are effective antibiotics, but they present significant risks, especially ototoxicity.

Tobramycin is also a promising natural antibiotic from the aminoglycoside group, and it is widely used for treating several bacterial infections [258]. This antibiotic is an excellent choice in the aminoglycoside group for treating serious infections caused by *Pseudomonas aeruginosa* due to its enhanced antibacterial activity against this pathogen. It can be delivered via several ways, including intravenous, intramuscular, inhaled, or ophthalmic use. For example, inhaled tobramycin can reduce bacterial load in sputum, improving lung function for patients with cystic fibrosis or bronchiectasis. In general, therapies with tobramycin have been clinically beneficial [259]. Moreover, if tobramycin is administrated by inhalation as soon as the initial infection is identified, it will lead to a decrease in morbidity and mortality [260]. However, similar to gentamicin or other aminoglycosides, tobramycin has been associated with ototoxicity and nephrotoxicity during clinical use. Also, tobramycin is more dynamic than gentamicin against *P. aeruginosa* and has a higher inhibitory file as well. In order to efficiently assess the general frequency of adverse effects of administrating tobramycin, broader clinical data are required. Despite their therapeutic effectiveness in the treatment of complex infections, they have a relatively weak binding affinity to prokaryotic rRNA, potentially causing serious adverse effects in patients [258]. In the study conducted by L. Terpstra et al. [262], the effects of administering a tobramycin inhalation solution for long periods of time have been analyzed through a multicenter randomized controlled trial that analyzed the safety and efficacy in patients with bronchiectasis who suffered from frequent, persistent infection. The general outcome, carried out as a multicenter randomized controlled trial, rated the effectiveness and safety of a daily dose of tobramycin inhalation for a period of over 12 months. The findings indicate that patients receiving tobramycin experienced a notable decrease in exacerbation frequency. The treatment proved to be safe and enhanced quality of life scores. Consequently, once-daily, long-term tobramycin inhalation could represent a viable therapeutic approach for patients with severe bronchiectasis. In the context of the chronic pulmonary infections caused by *P. aeruginosa,* Y-H. Gao et al. [261] conducted a study in which their objective was to rate the proportion of patients for eradicating *P. aeruginosa* in every group formed following the treatment with inhaled tobramycin alone or combined with oral ciprofloxacin [261]. This multicenter study, which is placebo-controlled, randomized 364 adults with isolated *P. aeruginosa* and assessed the efficacy and safety of tobramycin and the combination of tobramycin and ciprofloxacin. Patients were assigned to one or more treatments, comprising a 12-week phase and a 24-week follow-up [261]. The administration of this treatment led to a substantial proportion of the patients achieving a sustained eradication of *P. aeruginosa* between weeks 24 and 36, alongside enhanced lung function and reduced exacerbation, hospitalization, and healthcare costs, suggesting a suitable and favorable safety profile [261].

Thus, natural antibiotics generally have safety and toxicity concerns, despite their provision of therapeutic efficacy through their specific properties, and they can be used for treating bacterial infections [182]. Patient-related factors, such as age, gender, and allergy history should be taken into consideration before these natural products are administered, even for short periods of time [102]. In order to enhance these concerns of safety, efficacy, and toxicity, future research should cover the evaluation of dosing strategies, drug monitoring, and diagnostic methods as they are crucial for therapeutic outcomes, minimizing side effects, and ensuring the clinical translation of natural antibiotics [103].

## 6. Current Limitations and Challenges

Natural antibiotics have unique beneficial characteristics, making them a promising alternative to conventional antimicrobial agents; however, several limitations and challenges hinder their clinical translation and widespread application. Their efficacy remains inconsistent due to factors such as variable PKs [263], poor bioavailability [264], and challenges regarding finding novel discoveries [265]; moreover, there are limitations in their use in specific applications or treatments due to their limited tissue penetration [266]. Understanding these limitations and challenges is crucial for the development and therapeutic use of natural antibiotics in relation to antimicrobial resistance.

To overcome these limitations and to ensure the successful translation of natural antibiotics, a multi-faceted approach involving clinical, industrial, and regulatory strategies is essential, as summarized in Table 5.

### The Clinical Trial Pipeline

The clinical pipeline for natural-product-derived antibiotics is active [84] but limited to a few promising candidates. A notable example is teixobactin, a compound discovered from previously unculturable soil bacteria. While it is not yet used in human clinical trials, its unique mechanism of action—binding to lipid precursors in the bacterial cell wall—has shown exceptional potency against a broad range of Gram-positive bacteria, including MRSA, in preclinical studies [84]. A key challenge is the compound’s limited solubility, which has spurred the development of more soluble synthetic variants, known as “isobactins”, which have been designed for potential intravenous administration [267].

Another example is sutezolid, a semi-synthetic oxazolidinone, which has completed Phase 2 trials for treating drug-resistant tuberculosis. These trials demonstrated that sutezolid has a more favorable safety profile compared to the existing drug linezolid, with a lower risk of serious side effects like anemia and nerve damage, positioning it as a potentially safer alternative for long-term treatment regimens [268].

One of the current limitations in this field is related to the way in which these natural compounds are evaluated: typically, evaluation is achieved through measuring the PK and PD potency of the drug. Thus, these traditional indices (PK/PD), including MIC, have been used as a way of measuring antibiotic concentrations in biological fluids to guide dosing decisions [269]. Even though these PK–PD metrics have been used in the development of new perspectives for new antibiotics in several clinical applications, it is known that they possess a number of substantial drawbacks [270]. Certain limitations are associated with the characteristics of nonclinical experimental models and methodologies; others are developing from the use of the MIC as a measure of antibiotics’ antibacterial activities [271]. Some other limitations have arisen from the fact that bacterial responses over time are guided by complex systems of biological processes, such as natural growth and death rates, antibiotics-induced killing, and the potential regrowth of bacteria [270].

Developing novel antibiotics, nowadays, remains a significant challenge because of the understanding of bacterial permeability and the reliance on generic in vitro models that overlook the complexity of host and bacterial systems [103]. The current progress in the field of antibiotics has been monitored by the WHO, which emphasizes that relying on the development of new antibiotics is insufficient in combating antimicrobial resistance and that more investment is needed in research surrounding the issue as a whole [265]. Thus, antibiotic resistance is still a significant global threat that requires continuous intervention. One alternative for overcoming this challenge is the concept of antibiotic potentiation, which uses molecules or strategies that interfere with essential metabolic bacterial pathways. The progress of research into antimicrobial agents with multiple activities—inhibiting bacteria and enhancing the immune system—represents a potential strategy. Moreover, targeting bacterial repair mechanisms that are activated following antibiotic exposure could serve as a powerful approach for eliminating resistant pathogens [102]. In the study of M. Farha et al. [103], three main challenges were identified in the development of new antibiotics. One major barrier in antibiotic development is linked to the lack of understanding of the way in which small molecules penetrate bacterial cells. This problem is critical for Gram-negative bacteria, whose dual-membrane structures pose barriers to compound accumulation. Despite significant screening efforts, many promising agents fail to exhibit cell activity, highlighting the necessity for predictive models [103]. Another major obstacle in developing novel antibiotics is the widespread reliance on in vitro microbiological assays that fail to replicate the complexity of the host environment. As common antibiotic screening typically uses rich laboratory media, it ignores significant host factors, including immune responses, nutrient limitations, or tissue-specific conditions [103]. This undermines the predictive value of preclinical assays, limiting the potential for discovering novel therapeutics. The third challenge for discovering novel antibiotics, as described in M. Farha et al.’s study [103], is the underestimation of the complex bacterial cell systems that sustain bacterial viability. Drug-delivery approaches have a significant chance of failing due to the dense and interconnected genetic and metabolic systems within bacteria that enable adaptive responses and resistance to antibiotics [102]. Thus, system-based strategies are encouraged and needed to uncover therapeutically relevant vulnerabilities to significantly accelerate and enhance the efficacy of treatments [103].

A specific limitation of certain natural antibiotics, such as aminoglycosides (e.g., gentamycin or tobramycin), is their variable efficacy against a range of pathogens. While these compounds are often a first-line treatment for bacterial conjunctivitis due to their broad-spectrum activity against Gram-negative bacteria, clinical trials have shown inconsistencies in cure and bacterial eradication rates [182].

Furthermore, alternative treatments, including other natural antibiotics like polymyxin B and trimethoprim, or synthetic agents such as fluoroquinolones, have sometimes demonstrated superior performances [272].

Another significant challenge is patient adherence, as these ophthalmic solutions often require frequent dosing. Although they are generally biocompatible and well tolerated, aminoglycosides can cause localized ocular side effects, including irritation, edema, pruritus, or burning, which may further compromise treatment success [182].

Drug-delivery systems incorporated with natural antibiotics could also face some problems and challenges due to their unpredictable release profiles, degradation, limited solubility, or structural limitations in the carriers (nanoparticles, liposomes). For example, nanoparticles could represent a significant risk to human health, even though they are incorporated with excellent qualities. Metallic nanoparticles present some toxicological effects attributed to size, agglomeration, or surface changes [273], and this could influence the potential incorporation and targeting of natural antibiotics. Furthermore, metallic nanoparticles exhibit thermodynamic instability and possess irritant and carcinogenic properties [208]. Polymer nanoparticles could exhibit poor entrapment efficacy for hydrophilic natural compounds that can be leaked during the emulsification process. For example, the use of metallic nanoparticles could involve problems related to cytotoxicity, degradation, and scaled-up preparation [274]. As carriers for natural antibiotics, liposomes present several drawbacks, such as limited stability and low encapsulation efficiency, especially for hydrophobic antibiotics [229]. Lipid components are prone to degradation at specific temperatures and are susceptible to oxidative and hydrolytic breakdown [275].

Natural antibiotics represent a promising alternative for overcoming antimicrobial resistance due to their unique therapeutic characteristics [103]. However, natural products are limited by poor PKs, challenges in developing novel antibiotics, bacterial resistance mechanisms, and problems related to incorporation in drug-delivery systems, such as toxicity, instability, poor encapsulation efficacy, and the possibility of degradation [102]. To overcome these limitations, novel strategies are required, such as enhanced drug-delivery systems, the determination of optimal doses, advanced predictive models, and combined therapies [182]. In this way, with further research and clinical efforts, natural antibiotics could have significant potential to become safer, less toxic or nontoxic, and more efficient options for combating antimicrobial resistance.

## 7. Conclusions

In this review, we summarize the significance of natural antibiotics as alternatives in combating antimicrobial resistance. We highlighted various types of natural antibiotics, examined their complex mechanisms of action under different conditions, and explored their complex mechanisms of action in drug-delivery systems with an emphasis on safety, efficacy, and toxicity. Additionally, we discussed their current limitations and challenges that prevent clinical translation, including several issues related to pharmacokinetics, bacterial resistance mechanisms, and the drawbacks of delivery systems.

Natural antibiotics can be broadly classified as follows, according to their sources: animal-, bacteria-, fungal-, and plant-derived antimicrobials. The antimicrobials in each of these categories have specific potential for combating various microbial infections. Animal sources provide a rich variety of specific antimicrobial compounds, and include compounds derived from insects, including bee products, as well as substances derived from reptile and marine animals, such as snakes or crustaceans. Bacterial compounds are still one of the most used and prolific natural sources exhibiting antimicrobial activity, due to their extensive diversity and colonization capabilities. These include products such as vancomycin, orthoformimycin, kibdelomycin, myxovirecin, and teixobactin which exhibit potent activity against a wide range of Gram-positive bacteria or infectious agents, such as drug-resistant strains. Fungal antimicrobials also have significant potential for use as natural antimicrobial compounds. Some key antibiotics derived from this category, including mirandamycin, along with compounds from marine and terrestrial fungi, have proven to have excellent potential for antimicrobial activity against MRSA through several mechanisms. Plant-derived antibiotics are represented by three major classes: alkaloids, terpenoids, and polyphenols. These are critical in traditional medicine. Their antimicrobial activities and their synergistic effects against various bacteria have been proven and confirmed in a significant number of studies.

Natural antibiotics represent a powerful and versatile tool for destroying infections, as they are capable of targeting bacteria through multiple complex mechanisms. These natural antibiotics could be paired with traditional antibiotics; as a result, they can enhance treatment effectiveness by equipping drugs to disrupt biofilms, inhibit efflux pumps, and increase membrane permeability. Thus, these natural compounds reduce the chances of resistance developing. In this way, the applied synergistic strategies provide a promising approach for consolidating existing antibiotics and overcoming antimicrobial resistance, while also refining the drug-development process through the use of clinically known compounds.

While natural antibiotics such as penicillin, vancomycin, and gentamicin demonstrate substantial clinical efficacy, they are also associated with safety and toxicity concerns. Their therapeutic use necessitates careful consideration of patient-specific factors, including age, sex, and allergy history. Optimizing the safety and effectiveness of these compounds requires further research into improved dosing strategies, advanced drug monitoring, and enhanced diagnostic methods.

Despite their therapeutic potential, the widespread application of natural antibiotics is constrained by several factors, including variable pharmacokinetics, low bioavailability, and an incomplete understanding of complex bacterial behaviors. Traditional in vitro evaluation methods often fail to accurately predict clinical outcomes, highlighting a significant gap between laboratory findings and patient results. Furthermore, the integration of natural antibiotics into drug-delivery systems—such as nanoparticles, liposomes, or hydrogels—presents additional challenges, including potential toxicity, degradation issues, and poor drug encapsulation. Addressing these limitations through deeper research into bacterial systems and advanced delivery technologies is essential if we are to fully harness the potential of natural antibiotics in combating antimicrobial resistance. The future of natural antibiotics lies in their transition from a subject of academic interest to a scalable, reliable therapeutic reality. To achieve this, several key research directions are crucial. First, the field must shift towards targeted therapies that focus on bacterial resistance and virulence mechanisms, offering a genuinely novel therapeutic advantage. This involves exploring new compounds that target the highly conserved bacterial pathways that are essential for survival. Second, a critical future perspective involves prioritizing sustainable and scalable production approaches. This will require the application of advanced technologies like synthetic biology and “omics” approaches to engineer microbes for consistent and robust manufacturing. Finally, the move from traditional use to modern medicine necessitates rigorous clinical validation. This means subjecting natural compounds to thorough clinical trials to confirm their efficacy and safety, with clear regulatory frameworks being essential for their integration into clinical practice.

## Figures and Tables

**Table 1:** Summary of natural antimicrobials.

Natural Product	Source Organism	Type	Target Bacteria	In Vivo Efficacy (Murine Model)	Synergistic Combination (FIC ≤ 0.5)	Ref.
Thymol	Plant (*Thymus* spp.)	Phenolic compound	*S. typhimurium*, *S. aureus*, *E. coli*	Effective in animal models [14]	Ampicillin, tetracycline, penicillin, erythromycin, novobiocin	[16]
Carvacrol	Plant (*Oregano* spp.)	Phenolic compound	*S. typhimurium*, *S. aureus*	Effective in animal models [17]	Ampicillin, penicillin, bacitracin	[22]
Cinnamaldehyde	Plant (*Cinnamon* spp.)	Aldehyde	*E. coli*, *S. aureus*	Effective in animal models [86]	Ampicillin, tetracycline, penicillin, erythromycin, novobiocin	[130]
Allyl isothiocyanate	Plant (*Mustard* spp.)	Isothiocyanate	*S. pyogenes*	Effective in animal models [87]	Erythromycin	[131]
Nisin	Bacteria (*Lactococcus*)	Bacteriocin peptide	Gram-positive bacteria	Effective in food models [88]	With other preservatives	[132]
Antimicrobial peptides	Animals, plants, *fungi*	Peptide	Broad-spectrum (bacteria, fungi, viruses)	Effective in animal models [89]	With antibiotics	[133]
Essential oils	Plants (*various*)	Oil mixture	Broad-spectrum	Effective in food/animal models [90]	With antibiotics	[134]

**Table 2:** Nanoparticle-based antibiotic delivery systems.

Nanoparticle Type	Key Features and Advantages	Example System and Application
Polymeric nanoparticles	Enhanced stability and solubility: protects drugs from degradation and improves their bioavailability. Controlled and targeted release: can be engineered to release antibiotics over an extended period or at specific sites of infection. Versatility: can be made from various biodegradable polymers like PLGA and chitosan [212].	Chitosan–dextran sulfate nanocapsules: can be used to deliver ciprofloxacin, significantly prolonging its half-life and increasing concentration in tissues like the spleen and liver [213].
Lipid-based nanoparticles	Biocompatibility: composed of lipids, making them highly compatible with biological systems. Encapsulation: can encapsulate both hydrophilic and hydrophobic drugs. Biofilm penetration: their structure allows them to penetrate the protective matrix of bacterial biofilms [214].	Liposomes: encapsulating vancomycin to enhance its stability and bioavailability, particularly for treating infections in challenging environments. Solid lipid nanoparticles (SLNs): provide controlled release and enhance drug bioavailability [215].
Inorganic and metal-based nanoparticles	Intrinsic antimicrobial activity: some, like silver and gold, can directly disrupt bacterial membranes or generate reactive oxygen species (ROS), reducing the likelihood of resistance. Synergistic effects: can be combined with conventional antibiotics to restore efficacy against resistant strains [216].	Silver nanoparticles: can be used to combat biofilms by penetrating the matrix and targeting dormant bacteria. Gold nanoparticles: functionalized with antibiotics to increase activity against drug-resistant bacteria like MRSA [217].

**Table 3:** Advantages and limitations of drug-delivery systems incorporated with antibiotics.

Drug-Delivery Systems	Advantages	Limitations	Ref.
Nanoparticles	Improve antibiotic activity; allow targeted release; multiple drugs can be loaded; enhance microbial activity; improve stability potential for more accurate evaluation.	Size- and shape-dependent toxicity; poor intracellular penetration; increased surface area of NPs increases chemical reactivity, which leads to critical instability.	[187,197,209,211,218]
Hydrogels	Biocompatible; supports cell adhesion; provides sustained release of antibiotics; increases patient compliance; enhanced biofilm activity when combined with enzymes.	Slow responsiveness of stimuli-sensitive hydrogels; risk of burst release or incomplete release; possibility of drug deactivation.	[205,223,241]
Liposomes	Excellent biocompatibility; biodegradable; encapsulate both hydrophilic and hydrophobic drugs; improved wound healing; possess flexibility to couple with specific ligands.	Short shelf-life of lipid vesicles, limiting drug stability; aggregation and fusion of liposomal vesicles influence the efficacy of the drug; high production costs.	[206,226,229]
Solid lipid nanoparticles (SLNs)	Biocompatibility and biodegradability; controlled drug release profile; low toxicity; enhanced biofilm inhibition.	Low expulsion of drug time; limited ability to encapsulate hydrophilic drugs; drug expulsion during storage.	[238,242,243]

**Table 4:** Safety, efficacy, and toxicity of selected natural antibiotics.

Antibiotic	Class	Key Efficacy	Safety and Toxicity Concerns	Clinical Considerations	Ref.
Penicillin	β-lactam	Broad-spectrum activity against Gram-positive bacteria [201].	- Allergic reactions (1–10% of population, often over-diagnosed). - IgE-mediated hypersensitivity, which is rare (1–2% of adverse reactions) [202,203].	- Skin testing reliable for de-labeling allergies (especially in children). - Pharmacist-driven protocols improve accurate allergy assessment.	[247,248,249,250]
Vancomycin	Glycopeptide	Effective against MRSA and Gram-positive infections [205].	- Nephrotoxicity risk (especially in kidney disease or obesity). - Altered PKs in critically ill patients [206].	- Loading doses improve therapeutic levels without increasing toxicity. - Individualized dosing needed for obese/renal-impaired patients.	[251,252,253]
Gentamicin	Aminoglycoside	Synergistic with β-lactams; effective against Gram-negative bacilli [208].	- Nephrotoxicity and irreversible ototoxicity (higher risk in children and elderly and critically ill people). - Narrow therapeutic window [209,210].	- Once-daily dosing preferred (reduces toxicity). - Therapeutic drug monitoring (TDM) recommended for high-risk patients.	[254,255,256,257]
Tobramycin	Aminoglycoside	Superior anti-*Pseudomonas* activity (vs. gentamicin) [212].	- Ototoxicity and nephrotoxicity (similar to gentamicin). - Weak rRNA binding may increase side effects [213,214].	- Inhaled form reduces exacerbations in cystic fibrosis/bronchiectasis. - Long-term inhalation therapy improves quality of life.	[258,259,260,261,262]

**Table 5:** Key strategies for the successful incorporation of natural antibiotics in clinical and industrial settings.

Strategy	Description	Key Considerations	Ref.
Clinical protocols	Integrating natural antibiotics into standard treatment guidelines [201].	Optimizing dosing (e.g., vancomycin loading doses), mitigating toxicity risks, and implementing allergy de-labeling (e.g., penicillin skin testing).	[247,253]
Industrial scalability	Overcoming challenges in large-scale production and standardization [18].	Using biotechnological advancements like CRISPR-based strain engineering and “omics-driven” discovery to ensure stability and consistent quality.	[23,24]
Synergistic formulations	Developing combination therapies to enhance efficacy [79].	Pairing natural antibiotics (e.g., polyphenols) with conventional drugs (e.g., β-lactams) to increase effectiveness and delay resistance.	[123,160]
Regulatory and economic rules	Advocating for policies that support the development of natural antibiotics [198].	Highlighting their lower toxicity profiles and multi-target mechanisms to encourage investment and streamline approval processes.	[244,265]

## Data Availability

Not applicable.

## Abbreviations

The following abbreviations are used in this manuscript:

AMR	antimicrobial resistance
WHO	World Health Organization
MDR	multidrug-resistant
ESKAPE pathogens	*Enterococcus faecium*, *Staphylococcus aureus*, *Klebsiella pneumoniae*, *Acinetobacter baumannii*, *Pseudomonas aeruginosa*, and *Enterobacter* spp.
CRISPR	Clustered Regularly Interspaced Short Palindromic Repeats
AMPs	antimicrobial peptides
3D	three-dimensional
NPs	nanoparticles
MRSA	methicillin-resistant *Staphylococcus aureus*
H_2_O_2_	hydrogen peroxide
10-HDA	10-hydroxy-2-decenoic acid
CaTx-II	*Crotalus adamanteus* toxin-II
MIC	minimum inhibitory concentrations
MBC	minimum bactericidal concentrations
VRE	Vancomycin-resistant Enterococci
MTN	mesoporous titanium nanoparticles
PEI	ethylene imine polymer
BH	berberine hydrochloride
BER	berberine chloride
GBM	glioblastoma
PLGA	poly lactic-co-glycolic acid
ALG	blending alginate
MC	methylcellulose
LAP	laponite
Gen	gentamicin
PVA	Polyvinyl alcohol
MVLs	multivesicular liposomes
VAN HL	vancomycin hydrochloride
SLNs	solid lipid nanoparticles
NRG	naringenin
PM	paromomycin ulphate
VM-FB	vancomycin base
MSSA	methicillin-sensitive *Staphylococcus aureus*
PTA	target attainment
PK	pharmacokinetic
PD	pharmacodynamic
OHA-PBA	acid-grafted oxidized hyaluronic acid
SpsB	signal peptidase type IB
